# Efficacy and effectiveness of melatonin for the management of musculoskeletal pain: a systematic review and meta-analysis of placebo and active controlled trials

**DOI:** 10.1097/j.pain.0000000000004045

**Published:** 2026-06-30

**Authors:** Kangchao Wu, Ting Him Ho, Paula R. Beckenkamp, Michelle Hall, Hanzhi Zhang, James Puterflam, Karin Due Bruun, Jianhua Lin, Christopher Gordon, Ron Grunstein, Manuela L. Ferreira, Paulo Ferreira

**Affiliations:** a School of Health Science, Faculty of Medicine and Health, The University of Sydney, Sydney, NSW, Australia; b Chatswood Allied Health, Chatswood, Sydney, NSW, Australia; c Sydney Musculoskeletal Health, The Kolling Institute, University of Sydney, Sydney, NSW, Australia; d Musculoskeletal Research Hub, Charles Perkins Centre, The University of Sydney, Sydney, NSW, Australia; e Pain Research Group, Pain Center, Odense University Hospital, Odense, Denmark; f Department of Physiotherapy, Shanghai Yangzhi Rehabilitation Hospital (Shanghai Sunshine Rehabilitation Center), Tongji University School of Medicine, Shanghai, P. R. China; g CIRUS Centre for Sleep and Chronobiology, Woolcock Institute of Medical Research, Sydney, NSW, Australia; h Faculty of Medicine, Health and Human Sciences, Macquarie University, Sydney, NSW, Australia; i Royal Prince Alfred Hospital, Sydney, NSW, Australia; j The George Institute for Global Health, University of New South Wales, Sydney, NSW, Australia; k Exercise Medicine Research Institute, Edith Cowan University, Perth, Western Australia, Australia.

**Keywords:** Melatonin, Musculoskeletal pain, Postoperative pain, Sleep disturbance, Pain management, Meta-analysis

## Abstract

Melatonin may provide benefits for managing musculoskeletal (MSK) pain, and this review evaluated its efficacy and effectiveness on pain intensity and sleep quality. We searched 6 databases from inception to April 2025 and included randomized controlled trials in people with chronic or postoperative MSK pain reporting pain or sleep outcomes. Comparators included placebo or active controls (eg, analgesics). Random-effects meta-analyses were performed using mean differences with 95% confidence intervals. Subgroup analyses were conducted by comparator type. Sensitivity analyses included only low-risk studies. Certainty of evidence was assessed using GRADE. Twenty-three studies (2028 participants) were included. For people with chronic MSK pain, melatonin was not superior to placebo in reducing pain (mean difference [MD]: −6.76/100, 95% confidence interval [CI]: −15.89 to 2.36) but was superior to active controls (MD: −11.21/100, 95% CI: −14.29 to −8.13); in sensitivity analysis, melatonin was superior to placebo (MD: −10.04, 95% CI: −17.68 to −2.39). For people with postoperative pain, melatonin was superior to placebo, with effects similar to active controls. Sleep quality favoured melatonin in people with chronic MSK pain but not in those with postoperative pain. Adverse events were mild and transient. Melatonin may provide modest benefits for pain in people with chronic MSK pain, while evidence in postoperative pain remains limited. Although treatment effect estimates were comparable with conventional analgesics, most did not reach minimal clinically important difference thresholds and the certainty of evidence was low to moderate. Melatonin may be considered as a potential adjunct rather than a primary treatment, pending randomized controlled trials with standardised dosing and longer follow-ups.

## Abstract 2

Supplemental Digital Content is Available in the Text.

## 1. Introduction

Musculoskeletal (MSK) pain, including low back pain and osteoarthritis, affected up to 47% of the global population in 2020.^20^ Musculoskeletal pain negatively affects sleep, and vice versa—with this bidirectional relationship being consistently documented in the literature.^17,29,58^ Pain often causes discomfort and activates stress responses, making it harder to fall and stay asleep.^10^ Conversely, sleep disturbance heightens pain severity and pain sensitivity by increasing the concentration of substances (eg, interleukin-1β, interleukin-6, and tumor necrosis factor-alpha) that promote inflammation and disrupts central pain inhibitory mechanisms.^26^

Melatonin is an endogenous hormone produced by the pineal gland and is commonly used as an over-the-counter supplement for sleep problems, although clinical guidelines generally do not recommend it as a first-line treatment for insomnia.^28^ A 2013 systematic review of 19 trials (n = 1683) showed that melatonin reduced sleep latency and increased total sleep time compared with placebo in people with insomnia.^70^ Melatonin also seems to have a favourable safety profile. Existing reviews show that high dose or long-term use of melatonin does not lead to drug dependence or serious adverse events.^25,47^

Several animal studies conducted over the past decades have suggested that melatonin may have analgesic properties, potentially mediated through various neurotransmitter pathways.^3^ In humans, melatonin is currently being investigated for its analgesic effects in clinical trials.^38^ A 2025 systematic review of 8 trials (n = 1018) reported inconsistent and limited evidence regarding the postoperative analgesic effects of melatonin, and no meta-analysis was performed due to substantial heterogeneity in melatonin administration protocols and outcome measurements.^57^ Another systematic review of 30 trials (n = 1967) published in 2020 concluded that melatonin might be effective in reducing chronic pain but not in the treatment of acute postoperative pain; however, this review included all types of pain without specifically focusing on MSK pain.^52^ Other existing systematic reviews often report unreliable conclusions regarding melatonin's analgesic effects, or included heterogeneous study designs (eg, randomized controlled trials and nonrandomized controlled trials), making it difficult to isolate the effect of melatonin on MSK pain conditions.^4,30,67^ Moreover, to the best of our knowledge, no previous review has evaluated both melatonin's efficacy in placebo-controlled trials and effectiveness in active-controlled trials, representing an important gap in the field.

Thus, the aim of this systematic review and meta-analysis was to determine the effectiveness and efficacy of melatonin in randomized controlled trials (RCTs) on pain intensity and sleep quality in patients with chronic MSK pain and in patients undergoing surgery for MSK conditions.

## 2. Methods

This systematic review and meta-analysis was conducted according to the Preferred Reporting Items for Systematic Reviews and Meta-Analyses (PRISMA) and prospectively registered on PROSPERO (ID: CRD42023495805).^54^ This study did not require approval from an institutional ethics committee because of its nature as a systematic review.

### 2.1. Data sources and searches

We conducted searches for eligible studies across 6 electronic databases from inception until April 10, 2025, including the Cochrane Library, MEDLINE (Ovid), EMBASE (Classic and Ovid), PsycINFO (Ovid), CINAHL, and Web of Science (see Supplementary Material 1, http://links.lww.com/PAIN/C537 for details of the search strategy). We used an exhaustive list of keywords for “melatonin,” “musculoskeletal pain,” and related terms for various MSK pain conditions, such as fibromyalgia, knee osteoarthritis, neuropathy, low back pain, and postoperative pain. We also searched the reference list of relevant systematic reviews and included studies to identify additional search terms and publications. Identified references were imported to Covidence software (Veritas Health Innovation, Melbourne, Australia) and duplicates were removed.

### 2.2. Study selection and eligibility

Only RCTs involving humans were eligible. Studies in any language and with any follow-up duration were eligible for inclusion. No restrictions were applied on studies' publication dates.

Studies were included if they involved: (1) populations with a diagnosis or report of chronic MSK pain (defined as pain persisting for ≥3 months) including fibromyalgia, low back pain, neck pain, thoracic pain, rheumatoid arthritis, osteoarthritis, shoulder pain, pelvic pain, knee pain, and any other soft tissue injuries; (2) participants who had surgery for any MSK conditions including knee, shoulder, hip arthroplasty, fracture-related, and spinal surgeries; (3) studies had to include melatonin as a treatment in any dosage, any method of administration, alone or in conjunction with other medications or conservative treatments. Comparators could include placebo (for efficacy analysis) or other medications and forms of conservative care (for effectiveness analysis). To be included, studies had to include outcomes of pain intensity or sleep quality as primary or secondary outcomes. Studies were excluded if participants had a diagnosis of serious MSK pain pathologies including cancer, spinal infection, and cauda equina syndrome.

### 2.3. Study screening

Two reviewers (K.W., H.Z.) independently conducted the screening for eligibility in 2 separate stages using the Covidence software (Veritas Health Innovation). Titles and abstracts were initially screened, followed by full-text review. Discrepancies were resolved either by achieving a mutual agreement or with the involvement of a third reviewer (P.F.).

### 2.4. Data extraction

Two reviewers (K.W., H.Z.) independently extracted data using a prepiloted, standardized excel spreadsheet. Disagreements were resolved through consensus. Population characteristics (eg, age, sex), melatonin dose regimen, and pain and sleep outcome measures were extracted. When multiple measurement scales were reported within the same domain, we prioritized the visual analogue scale (VAS) for pain outcome, and the Pittsburgh Sleep Quality Index (PSQI) for sleep outcome. These outcomes were selected for their high reproducibility and reliability.^11,14^ For studies not using VAS and using more than one measurement for the pain outcome, we preferred the Knee injury and Osteoarthritis Outcome Score (KOOS) pain subscale over the Short Form-36 (SF-36) pain component or the Lequesne Index pain component in Shodiev 2023^59^ because postintervention pain data were available only for the KOOS pain subscale. For studies not using PSQI and using more than one measurement for the sleep outcome, we preferred the Subjective Sleep Quality Scale (SQS) over the Stanford Sleepiness Scale (SSS) in Kirksey 2015^39^ and the Patient-Reported Outcomes Measurement Information System (PROMIS) sleep disturbance form over the Epworth Sleepiness Scale (ESS) in Haider 2024.^27^ This is because SQS and PROMIS capture nocturnal sleep quality, which aligns with the predefined sleep outcome of this review, whereas SSS and ESS reflect daytime sleepiness. When trials had multiple treatment arms of melatonin using different dosages, we extracted data from the arm with the highest dosage. Continuous outcomes measured using different scales (eg, VAS and numeric rating scale [NRS] for pain intensity) were converted to a standardized 0 to 100 scale.^50^

When standard deviations (SDs) were not reported, they were calculated from standard error (SE) (SD = SE × √n) or 95% confidence interval [CI] (SD = √n × (upper − lower)/3.92). Medians and interquartile range (IQR) were converted to means (SDs) using mean ≈ median, SD ≈ (P75 − P25) × 0.7413.^31^

If the raw data were not reported in the text, we attempted to contact the corresponding authors to obtain the missing information. If figure data were absent from the text and could not be obtained from the corresponding author, Plot Digitiser was used to extract data from graphs for meta-analysis.^1,21,23,42^ This approach is commonly used in systematic reviews when numerical data are unavailable, and methodological studies suggest that extraction error is unlikely to meaningfully affect effect estimates.^7,63^

### 2.5. Quality assessment

Study methodological bias was assessed using the Revised Cochrane risk of bias tool for RCTs (RoB 2.0), performed independently by 2 reviewers (K.W., H.Z.).^62^ Discrepancies were then resolved by mutual agreement or the involvement of the third reviewer (P.F.).

### 2.6. Data synthesis and analysis

Meta-analysis was performed on the effectiveness of melatonin compared with active controls (eg, analgesic medications), and on the efficacy in placebo RCTs using software Review Manager 5.4.^31^ We used weighted mean differences with 95% CIs to summarize mean values or mean changes. A random-effects model meta-analysis (DerSimonian and Laird method) was used, as the included trials were conducted in different populations and involved variations in melatonin administration. The I^2^ statistic was used to quantify heterogeneity among the included RCTs, with values greater than 50% considered indicative of substantial heterogeneity.^32^

We conducted separate meta-analyses for chronic MSK pain and postoperative MSK pain. Within each population, subgroup analyses were performed for each outcome according to comparator type (placebo, analgesic medications, or nonanalgesic medications) and surgical condition, to explore potential sources of heterogeneity. We used the immediate post-treatment outcome from each study for meta-analysis, as this time point was considered most appropriate to capture the effect of melatonin at the completion of treatment and to improve comparability across studies.^12^ In postoperative studies where melatonin was administered only once, we selected the time point at which outcome was assessed closest to 24 hours after surgery for analysis. Although we attempted to extract data for medium (>3 months but <12 months) and long-term (≥12 months) follow-ups, only one trial reported outcomes at medium-term time points (4 and 6 months),^56^ while all others provided short-term follow-up data only. Sensitivity analyses were conducted to assess the robustness of the findings by including only studies with low risk of bias. Exploratory meta-regression analyses were conducted, where possible, to examine whether study-level intervention characteristics (eg, melatonin dose, treatment duration, and number of administrations) were associated with effect estimates. Publication bias was assessed by inspecting the asymmetry of funnel plots, and performing the Egger test when a comparison included more than 10 studies.^43,60^

### 2.7. Certainty of evidence

To evaluate the certainty of evidence, Grading of Recommendations Assessment, Development, and Evaluation (GRADE) was performed by 2 independent reviewers (K.W., H.Z.) using the software GRADEpro GDT (McMaster University and Evidence Prime, 2024). The risk of bias was downgraded if more than half of the participants were from high risk of bias studies. Inconsistency was downgraded if substantial unexplained heterogeneity (I^2^ > 50%) was identified. Imprecision was downgraded if the calculated optimal information size was not met. Indirectness was downgraded if the population, intervention, comparator, or outcomes were not directly aligned with the research question (eg, pain reported as part of a composite outcome, or population not representative of chronic MSK pain). Publication bias was downgraded if significant publication bias was identified using the Egger regression test (*P* < 0.05) and visual inspection of funnel plot asymmetry.

## 3. Results

### 3.1. Study selection

**Figure 1.**
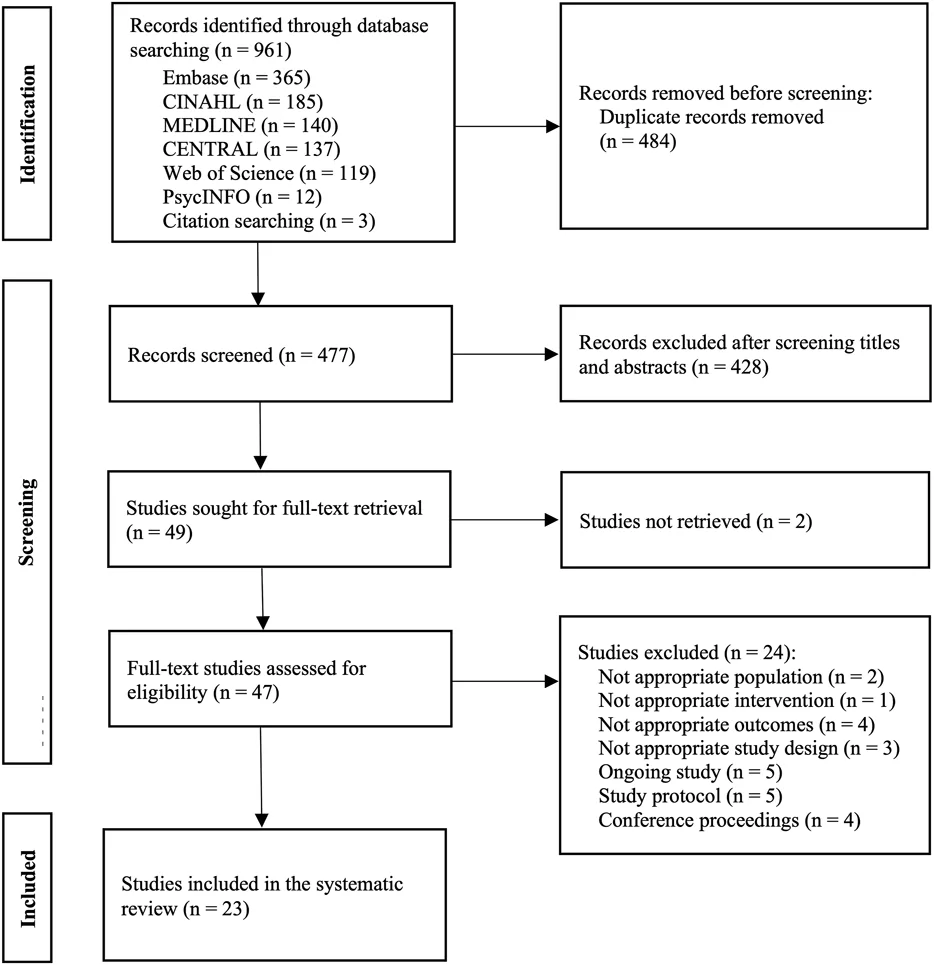
PRISMA flow diagram of the literature search.

Our search identified 961 records from the 6 databases. After removing duplicates, 477 titles and abstracts were screened, followed by 49 full texts. In total, 23 studies were included in this review (Fig. 1).

### 3.2. Study characteristics

The 23 RCTs included a total of 2028 participants with a diagnosis of chronic MSK pain or postoperative MSK pain.^1,2,5,9,18,19,21,23,26,33–36,39,41,42,48,49,54,55,58,64,68^ All studies used a parallel-arm RCT design including 14 placebo-controlled trials. Seven trials were conducted in Iran,^9,19,34,35,48,49,55^ 5 in United States,^18,27,39,42,56^ 3 in Russia,^36,41,59^ 2 in Brazil,^64,68^ 2 in Egypt,^1,21^ 1 in China,^23^ 1 in Iraq,^33^ 1 in Turkey,^2^ and 1 in India.^5^ Melatonin was administered orally in all studies, except one study where the drug was administered through transdermal patch.^21^ Treatment duration ranged from a single administration to 90 days, with dosages ranging from 1 mg to 10 mg.

Postoperative pain was investigated in 14 studies,^1,5,9,18,21,23,27,34,35,39,42,48,49,55^ primarily involving joint replacement and spinal surgeries. Chronic MSK pain was investigated in 9 studies,^2,19,33,36,41,55,59,64,68^ including pain conditions such as fibromyalgia, knee osteoarthritis, and low back pain. More details on the characteristics of the included trials and the medications evaluated are provided in the supplementary table (Table S1, http://links.lww.com/PAIN/C537).

### 3.3. Risk of bias

The overall risk of bias among the included studies was judged to be low to moderate (Fig. S1, http://links.lww.com/PAIN/C537). Most studies reported an appropriate method for random sequence generation and allocation concealment. Blinding was appropriately implemented in most studies, with participants, intervention providers, and outcome assessors blinded in 20 of 23 studies. Four trials (17%) reported dropout rates exceeding 15% and were considered to have high dropout rates. The detailed risk of bias assessment for each individual trial is presented in supplementary figure S2, http://links.lww.com/PAIN/C537.

### 3.4. Effectiveness and efficacy of melatonin on reducing chronic musculoskeletal pain

**Figure 2.**
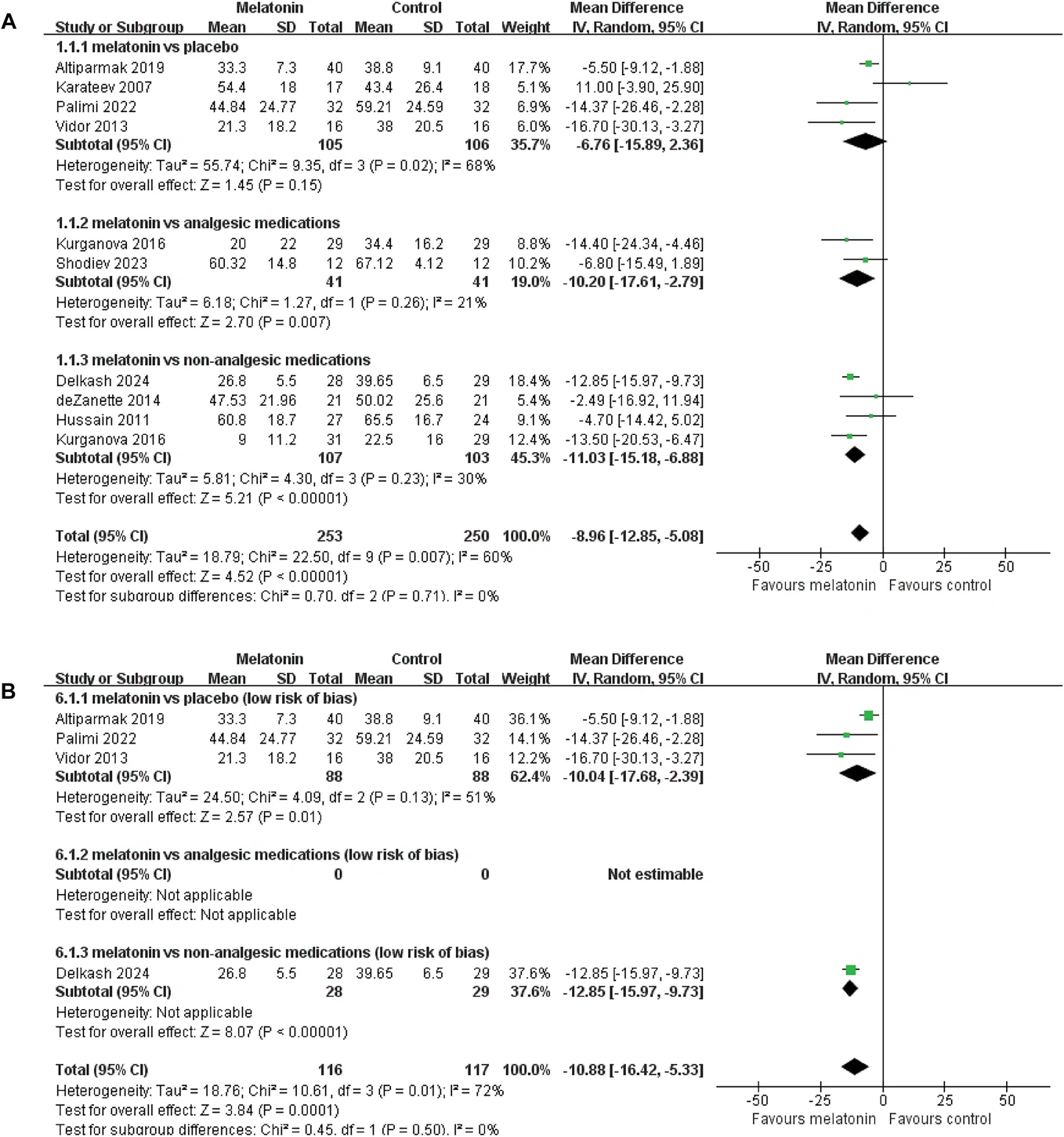
Forest plot of the effect of melatonin on pain intensity in patients with chronic musculoskeletal pain. (A) shows the primary meta-analysis; (B) shows the sensitivity analysis restricted to studies with low risk of bias. Pain scores are reported on a 0 to 100 scale. CI, confidence interval; SD, standard deviation.

Figure 2A shows the results of the meta-analysis pooling 9 studies investigating the effect of melatonin on pain intensity in individuals with chronic MSK pain.^2,19,33,36,41,55,59,64,68^ Using a random-effects model, melatonin was associated with a significant reduction in pain compared with all comparator treatments, with a pooled mean difference (MD) of −8.96/100 (95% CI: −12.85 to −5.08; *P* < 0.0001; I^2^ = 60%). The evidence was low certainty according to GRADE guidelines and downgraded due to risk of bias and serious inconsistency.

When compared with placebo, melatonin reduced pain by a mean difference of −6.76/100 (95% CI: −15.89 to 2.36; *P* = 0.15), but this reduction was not statistically significant (Fig. 2A). By contrast, melatonin demonstrated a significant reduction in pain when compared with analgesic medications such as diclofenac (MD: −10.20, 95% CI: −17.61 to −2.79; *P* = 0.007), as well as when compared with nonanalgesic medications such as fluoxetine and amitriptyline (MD: −11.03, 95% CI: −15.18 to −6.88; *P* < 0.0001).

The sensitivity analysis included 4 studies with low risk of bias and showed that melatonin intake resulted in significant reductions in pain against all comparators, with a pooled mean difference of −10.88/100 (95% CI: −16.42 to −5.33; *P* = 0.0001; I^2^ = 72%) (Fig. 2B). Notably, the reduction in pain scores was significant when compared with placebo (MD: −10.04, 95% CI: −17.68 to −2.39; *P* = 0.01), and the heterogeneity within this subgroup decreased from I^2^ = 68% in the primary analysis to I^2^ = 51%. The effect of melatonin on pain remained significant when compared with nonanalgesic medications (MD: −12.85, 95% CI: −15.97 to −9.73; *P* < 0.0001). No studies with low risk of bias were available in the analgesic comparator group.

### 3.5. Effectiveness and efficacy of melatonin on reducing postoperative musculoskeletal pain

**Figure 3.**
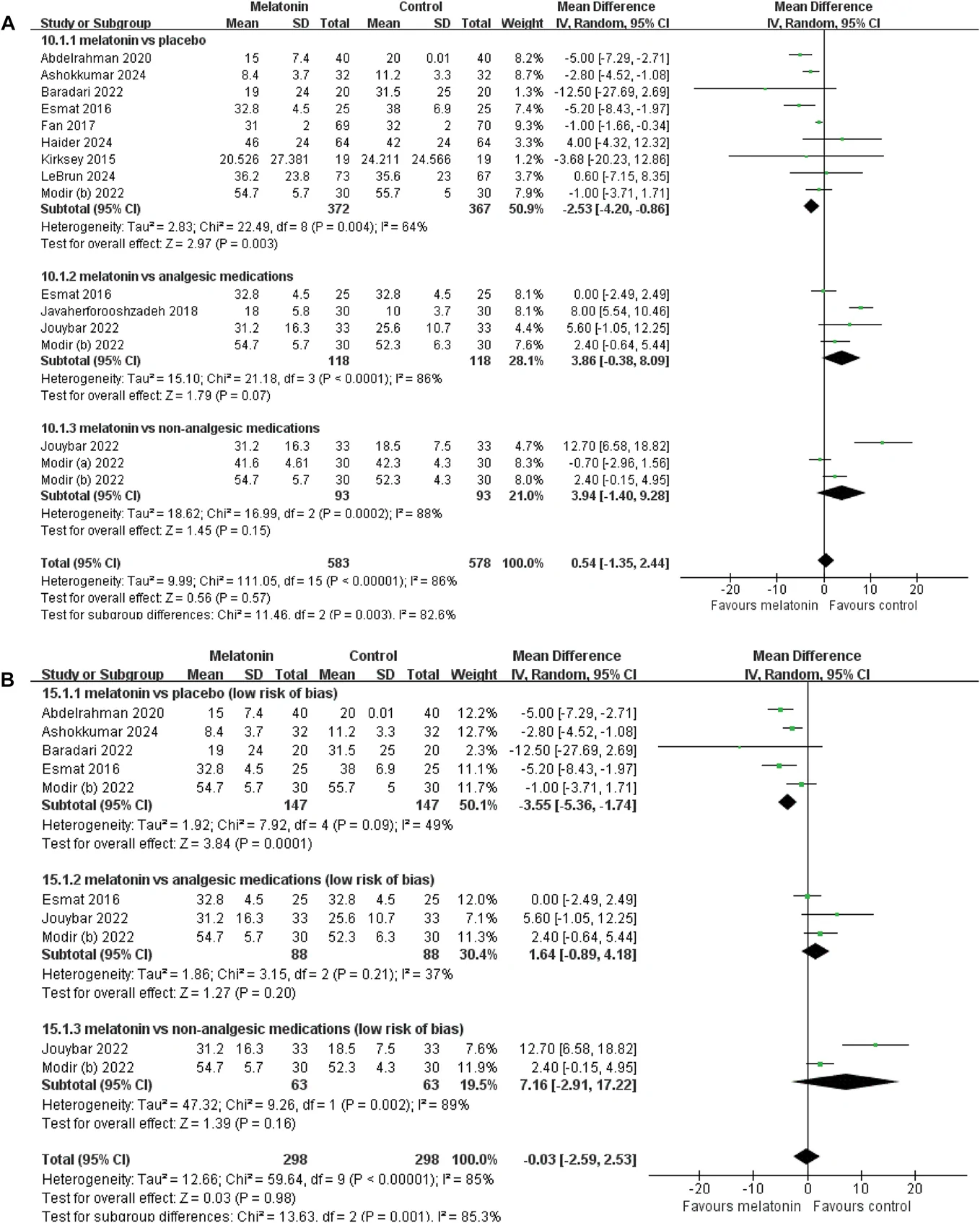
Forest plot of the effect of melatonin on pain intensity in patients with postoperative musculoskeletal pain. (A) shows the primary meta-analysis; (B) shows the sensitivity analysis restricted to studies with low risk of bias. Pain scores are reported on a 0 to 100 scale. CI, confidence interval; SD, standard deviation.

Figure 3A presents the meta-analysis of 12 studies investigating the effect of melatonin on postoperative pain.^1,5,9,21,23,27,34,35,39,42,48,49^ Overall, no significant difference was observed between melatonin and all comparator treatments, with a pooled mean difference of 0.54/100 (95% CI: −1.35 to 2.44; *P* = 0.57; I^2^ = 86%). The evidence was moderate certainty according to GRADE guidelines and downgraded due to serious inconsistency.

In the subgroup analysis based on comparator type, melatonin was found to significantly reduce postoperative pain when compared with placebo (MD: −2.53, 95% CI: −4.20 to −0.86; *P* = 0.003) (Fig. 3A). However, when compared with analgesic medications (eg, fentanyl, gabapentin), the reduction in pain favored the analgesic medication group, albeit not statistically significant (MD: 3.86, 95% CI: −0.38 to 8.09; *P* = 0.07). Similarly, the pain reduction favored nonanalgesic medications (eg, dexmedetomidine, dextromethorphan) albeit not statistically significant (MD: 3.94, 95% CI: −1.40 to 9.28; *P* = 0.15). Subgroup analyses by surgical condition are shown in the supplementary figure S3, http://links.lww.com/PAIN/C537.

The sensitivity analysis included 6 studies with low risk of bias and showed no significant difference in pain intensity between melatonin and all comparator treatments (MD: −0.03/100, 95% CI: −2.59 to 2.53; *P* = 0.98; I^2^ = 85%), consistent with the primary analysis (Fig. 3B). Melatonin was associated with a statistically significant reduction in pain compared with placebo (MD: −3.55, 95% CI: −5.36 to −1.74; *P* = 0.0001), with a moderate decrease in heterogeneity (I^2^ = 47% compared with 64% in the primary analysis). However, no significant differences were found when melatonin was compared with either analgesic medications (MD: 1.64, 95% CI: −0.89 to 4.18; *P* = 0.20; I^2^ = 37%) or nonanalgesic medications (MD: 7.16, 95% CI: −1.29 to 15.62; *P* = 0.10; I^2^ = 89%). Notably, the heterogeneity in the analgesic subgroup decreased markedly compared with the main analysis (I^2^ = 37% compared with 86% in the primary analysis), while the nonanalgesic subgroup remained highly heterogeneous (I^2^ = 89% compared with 88% in the primary analysis).

### 3.6. Effects of melatonin on sleep quality in chronic and postoperative musculoskeletal pain

**Figure 4.**
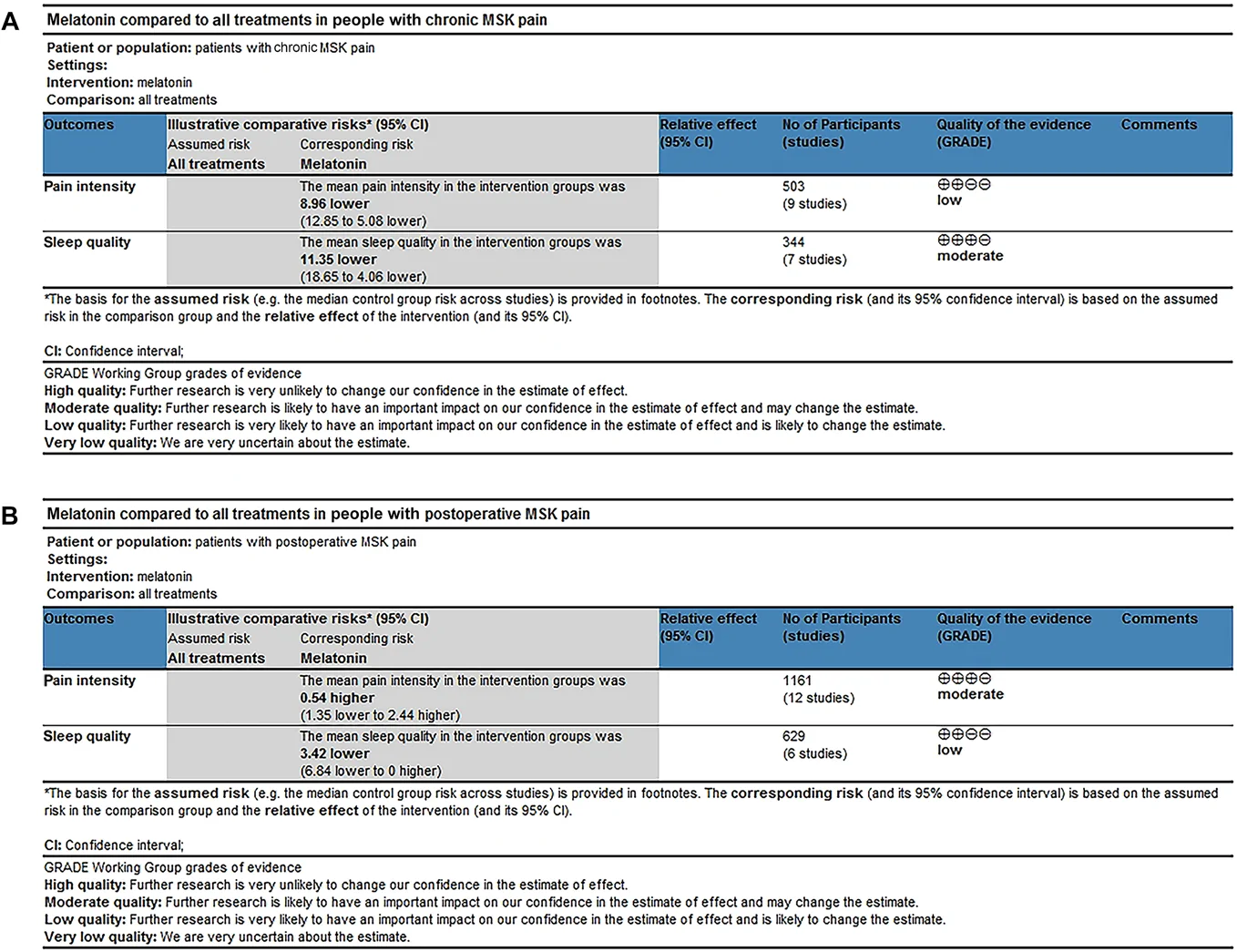
GRADE assessment for certainty of evidence on melatonin's effects on pain and sleep outcome in chronic (A) and postoperative (B) musculoskeletal pain. Results are shown as mean differences (0-100 scale) with 95% confidence intervals. CI, confidence interval; MSK, musculoskeletal.

Melatonin was associated with improved sleep quality compared with all treatments in participants with chronic MSK pain (MD: −11.35/100, 95% CI: −18.65 to −4.06; *P* = 0.002; I^2^ = 92%; 7 studies) (Fig. S4, http://links.lww.com/PAIN/C537). One study was excluded because the PSQI questionnaire was reported using a nonstandard scoring method (total score >21), preventing valid conversion for meta-analysis.^68^ The evidence was considered moderate quality due to serious inconsistency (Fig. 4A).

In postoperative populations, melatonin showed no significant difference in sleep improvement compared with all treatments (MD: −3.42/100, 95% CI: −6.84 to 0.00; *P* = 0.05; I^2^ = 64%; 6 studies) (Fig. S5, http://links.lww.com/PAIN/C537). The evidence was considered low quality due to serious risk of bias and inconsistency (Fig. 4B).

### 3.7. Melatonin dose and duration: descriptive summary and exploratory meta-regression

Among trials in chronic MSK pain, melatonin doses ranged from 3 mg/day to 10 mg/day. Beneficial effects were observed with 3 mg/day in patients with chronic low back pain, rheumatoid arthritis, and neuropathic pain, with 5 mg/day in temporomandibular disorders, and with titrated dosing from 3 mg/day up to 10 mg/day in knee osteoarthritis (Fig. S6, http://links.lww.com/PAIN/C537). In postoperative MSK pain, trials showing benefit used doses ranging from 1 mg/day to 10 mg/day, including 1 mg/day in hip arthroplasty, 5 mg/day in patients with multiple rib fractures, 7 mg/day in lumbar laminectomy, and 10 mg/day in zygomaticomaxillary complex fractures (Fig. S7, http://links.lww.com/PAIN/C537). Across these studies, treatment duration and number of administrations varied substantially. In chronic MSK pain, treatment duration ranged from 4 weeks to 3 months (Fig. S8, http://links.lww.com/PAIN/C537). In postoperative MSK pain, regimens ranged from single-dose to 15 times (Fig. S9, http://links.lww.com/PAIN/C537).

To further explore whether dosage or treatment duration influenced treatment effects, exploratory meta-regression analyses were conducted (Table S2, http://links.lww.com/PAIN/C537). In chronic MSK pain, melatonin dose was not significantly associated with the treatment effect size (β = 0.83, 95% CI −2.22 to 3.88, *P* = 0.547), whereas longer treatment duration was associated with more favourable effects (β = −0.97, 95% CI −1.62 to −0.32, *P* = 0.009). In postoperative pain, neither melatonin dose (β = 0.38, 95% CI −0.81 to 1.57, *P* = 0.506) nor number of administrations (β = −0.08, 95% CI −0.38 to 0.23, *P* = 0.606) was significantly associated with the treatment effect size. These findings suggest no clear dose–response relationship in either chronic or postoperative MSK pain, while longer treatment duration may be associated with greater benefit in chronic MSK pain.

### 3.8. Publication bias

Publication bias was assessed only for the comparison of melatonin vs all treatments in postoperative conditions, as this was the only analysis including more than 10 studies. Visual inspection of the funnel plot suggested symmetry, indicating no apparent risk of publication bias (Fig. S10, http://links.lww.com/PAIN/C537). The Egger test also showed no statistically significant evidence of publication bias (*P* = 0.687).

### 3.9. Adverse events

Among the 23 included trials, 14 reported on adverse events.^1,5,9,21,23,35,36,39,42,48,49,54,64,68^ Nausea/vomiting were found in 7 studies (50%),^1,9,21,23,35,39,42^ with an occurrence rate of 20.5% (53/259) in the melatonin group. Dizziness was found in 4 studies (28.6%),^1,21,23,39^ with an occurrence rate of 15.0% (23/153) in the melatonin group. Headache was reported in 3 studies (21.4%),^23,42,55^ with an occurrence rate of 5.9% (11/187) in the melatonin group. Drowsiness was reported in 2 studies (14.3%),^1,42^ with an occurrence rate of 5.6% (7/126) in the melatonin group. All adverse events are summarized in Table 1.

**Table 1:** Adverse events.

Author, y	Reported adverse events	No. of adverse events			
		Melatonin (n/N)	%	Control (n/N)	%
Abdelrahman, 2020^1^	Nausea and vomiting	21/40	52.5%	6/40 (Placebo)	15%
	Bradycardia	1/40	2.5%	1/40 (Placebo)	2.5%
	Dry mouth	1/40	2.5%	0/40 (Placebo)	0%
	Drowsiness and dizziness	1/40	2.5%	0/40 (Placebo)	0%
Altiparmak, 2019^2^	NR	NR	NR	NR	NR
Ashokkumar, 2024^5^	Not specified	0/32	0%	NR	NR
Baradari, 2022^9^	Vomiting	3/20	15%	2/20 (Placebo)	10%
Clarkson, 2022^18^	NR	NR	NR	NR	NR
de Zanette, 2014^68^	Not specified (AEs defined as: dry mouth, severe dizziness, vivid nightmares, crippling drowsiness, severe headache, behavioural changes, pain worsening)	2/21	9.5%	2/21 (Amitriptyline)	9.5%
Esmat, 2016^21^	Dizzy	2/25	8%	N/A	N/A
	Nausea	0/25	0%	2/25 (Placebo)	8%
	Nausea and vomiting	0/25	0%	6/25 (Fentanyl)	24%
Fan, 2017^23^	Dizziness	11/69	15.9%	8/70 (Placebo)	11.4%
	Headache	8/69	11.6%	6/70 (Placebo)	8.6%
	Paraesthesia	9/69	13%	7/70 (Placebo)	10%
	Nausea	14/69	20.3%	12/70 (Placebo)	17.1%
Haider, 2024^27^	NR	NR	NR	NR	NR
Hussain, 2011^33^	NR	NR	NR	NR	NR
Jouybar, 2022^35^	Nausea	0 (0-1)*	N/A	1 (1-2)* (Dexmedetomidine) 2 (0-2)* (Gabapentin)	N/A
	Vomiting	0 (0-1)*	N/A	1 (1-1)* (Dexmedetomidine) 1 (0-2)* (Gabapentin)	N/A
Modir(b), 2022^27^	Not specified (AEs defined as: chills, nausea, vomiting, decreased consciousness)	0/30	0%	0/30 (Gabapentin)	0%
				3/30 (Dextromethorphan)	10%
				0/30 (Placebo)	0%
Delkash, 2023^19^	NR	NR	NR	NR	NR
Javaherforooshzadeh, 2018^34^	NR	NR	NR	NR	NR
Karateev, 2007^36^	Skin allergic	2/17	11.8%	0/18 (Placebo)	0%
Kirksey, 2015^39^	Dizziness	9/19	47.4%	7/18 (Placebo)	38.9%
	Nausea and vomiting	11/19	57.9%	6/18 (Placebo)	33.3%
Kurganova, 2016^41^	NR	NR	NR	NR	NR
Lebrun, 2024^42^	Drowsiness	6/86	7%	6/86 (Placebo)	7%
	Headache	1/86	1.2%	2/86 (Placebo)	2.3%
	Nausea	4/86	4.7%	0/86 (Placebo)	0%
Modir(a), 2022^48^	Not specified (AEs defined as: hypoxia, hypotension, bradycardia, shivering, nausea, vomiting, decreased consciousness)	Only reported no significant between-group difference; zolpidem was used as the control group			
Palimi, 2022^55^	Headache	2/32	6.25%	0/32 (Placebo)	0%
Perez, 2024^56^	NR	NR	NR	NR	NR
Shodiev, 2023^59^	NR	NR	NR	NR	NR
Vidor, 2013^64^	Not specified (AEs defined as: changes in mood, sleepiness, dizziness, headache, allergic reaction)	Only reported no serious or moderate AEs were observed in both group; placebo was used as the control group			

* Values reported as median (IQR) in this study, but the definition was not specified.

AE, adverse event; N/A, not applicable; NR, not reported.

## 4. Discussion

### 4.1. Summary of findings

In our meta-analysis, melatonin was found to be effective in reducing pain intensity and improving sleep quality across various chronic MSK pain conditions, regardless of the comparator type. Sensitivity analyses restricted to studies with low risk of bias further confirmed melatonin's efficacy, revealing a significant pain reduction compared with placebo. By contrast, melatonin showed no significant difference in reducing pain intensity or improving sleep quality in postoperative pain conditions across all comparators. Although a slight but statistically significant reduction in pain (−2.53 mm on a 0-100 mm scale) was observed in the placebo efficacy analysis, the mean difference did not reach the recommended minimal clinically important difference (MCID) of 9.9 mm for acute postoperative pain.^51^

### 4.2. Potential mechanisms of melatonin for pain relief

The analgesic effects of melatonin are multifactorial. It modulates opioid and GABAergic neurotransmission and engages MT2 receptors, with experimental evidence showing that MT2 receptor activation reduces neuronal excitability and alleviates allodynia.^65^ In parallel, melatonin exerts antioxidant and anti-inflammatory effects by scavenging free radicals and suppressing proinflammatory mediators such as cytokines and prostaglandins.^22,65^ These antioxidant and anti-inflammatory properties mitigate oxidative stress and inflammatory process that contribute to persistent pain states. Together, these actions may attenuate central sensitisation and inflammation underlying chronic pain. Another potential mechanism is melatonin's indirect analgesic effect through the restoration of disrupted circadian rhythms, which are frequently linked to chronic pain.^16^ By adjusting circadian phase, melatonin enhances its inherent adaptive properties, such as antioxidant, anti-inflammatory, and immunomodulatory effects, that contribute to pain reduction.^66^ In addition, improved sleep quality and reduced anxiety, both mediated by melatonin, may further attenuate pain perception.^15^

Multiple studies indicate that in chronic MSK pain populations, sleep-targeted interventions are often accompanied by improvements in pain.^46,61^ This may represent another mechanism through which melatonin exerts analgesic effects. Consistent with this pattern, our review found that melatonin significantly improved sleep quality in individuals with chronic MSK pain, with parallel reductions in pain intensity. As a well-documented sleep aid, melatonin may contribute to pain reduction not only by restoring sleep but also by facilitating broader psychosocial and lifestyle improvements—including enhanced mood, reduced stress, increased physical activity and exercise, and healthier dietary patterns—that collectively reduce systemic inflammation and, in turn, alleviate pain.^40^

### 4.3. Comparison with previous studies

A 2017 meta-analysis of 19 RCTs reported that melatonin significantly reduced pain intensity both compared with all treatments combined and in subgroup analyses of operation-associated pain, inflammatory pain, and procedural pain.^70^ However, this review did not include a placebo-controlled subgroup to evaluate efficacy, and it used the Jadad scale for quality assessment, which is not the tool recommended by the Cochrane Collaboration.^31^ A subsequent meta-analysis of 30 RCTs published in 2020 concluded that melatonin was effective for chronic pain but ineffective for acute postoperative pain.^52^ However, this study included all types of pain and lacked a specific focus on MSK pain. In our study, a sensitivity analysis based on study quality was performed to assess the robustness of the findings. The results revealed that melatonin was superior to placebo in chronic MSK pain conditions, in contrast to the primary analysis without restricting study quality, which showed no significant benefits over placebo. In addition, although our primary meta-analysis showed significant pain reduction favouring melatonin compared with both analgesic medications (eg, nonsteroidal anti-inflammatory drugs [NSAIDs]) and nonanalgesic medications (eg, fluoxetine), no studies with low risk of bias were available for the analgesic comparison, and only one study was available for the nonanalgesic comparison. Therefore, the apparent superiority of melatonin over other medications should be interpreted with caution due to the low methodological quality of the included studies. These findings highlight the need for more high-quality studies comparing melatonin with active controls such as analgesic medications.

### 4.4. Implications of study findings

In the current meta-analysis, we identified an effect size of −8.96/100 for pain reduction when melatonin was compared with all treatments combined in patients with chronic MSK pain. Interestingly, this effect size is comparable with that of NSAIDs (MD: −7.7, 95% CI: −11.4 to −4.1; 2611 participants) for MSK pain, which are consistently recommended as a first- or second-line treatment option.^45,69^ Nevertheless, melatonin has several advantages. First, melatonin is inexpensive and affordable. In Australia, the cost is approximately 0.75∼1.5 Australian dollar per tablet.^13^ Second, melatonin seems to have a safer profile than other commonly used analgesics. In our systematic review, adverse events were infrequent and occurred at rates comparable with placebo comparisons. Other commonly used analgesics for MSK pain, such as paracetamol and opioids, are not recommended in most current clinical guidelines because of their limited effect, side effects, or the risk of dependency and addiction.^6,8,44,53^ Despite these advantages, this comparison with NSAIDs should not be interpreted as therapeutic equivalence, and the clinical applicability of melatonin remains uncertain. Although some pooled effects were statistically significant, they were not necessarily clinically meaningful, as many did not reach the accepted MCID threshold for pain intensity in chronic pain populations (11-14 mm).^37^ In addition, the certainty of evidence was low, further limiting confidence in the clinical applicability of these findings. Taken together, given its modest effects, affordability, and favourable safety profile, melatonin may be more appropriately considered as a potential adjunct option for MSK pain rather than a primary treatment.

Moving forward, several clinical factors should be considered and tested to refine the use of melatonin for both chronic and postoperative MSK pain. Key aspects include the optimal dose, dosing regimen, and duration of administration. Based on the included trials, melatonin doses ranged from 3 to 10 mg/day in chronic MSK pain and from 1 to 10 mg/day in postoperative MSK pain. Exploratory meta-regression analysis showed that melatonin dose was not significantly associated with treatment effect in either chronic or postoperative MSK pain, suggesting that a clearly optimal dose cannot be determined from the current evidence. By contrast, in chronic MSK pain, longer treatment duration was associated with more favourable effects, although this finding should be interpreted cautiously given the small number of studies. From a clinical perspective, commonly used doses (eg, 3 or 5 mg/day in chronic MSK pain and 5, 6, or 10 mg/day in postoperative pain) may be considered reasonable starting points, but dose standardisation and justification remain lacking in the included trials. In addition, evidence on higher doses of melatonin treatment (≥10 mg/day) for MSK pain remains limited, despite the safety of higher-dose melatonin having been established in a 2022 systematic review.^47^ This represents an important area for future research to determine whether higher doses confer additional benefit over commonly used dosing ranges. Future high-quality RCTs are needed to establish optimal treatment strategies, including dose, treatment duration, number and frequency of administration.

### 4.5. Strengths and limitations

The strengths of this systematic review are reflected in its restriction of the analyses to RCTs only, the focus on MSK pain conditions, the incorporation of placebo comparisons for efficacy analysis, and the separation of treatment comparators in effectiveness analyses between analgesic and nonanalgesic medications. Our study also has limitations that should be acknowledged. First, most included trials had a small sample size. The mean sample size of the included trials was 50 participants among chronic pain trials and 83 participants among postoperative pain trials. Second, most studies included in this meta-analysis lacked medium- to long-term follow-up data beyond 3 months. Future RCTs with extended follow-up periods are warranted to better establish the long-term effects of melatonin. Third, the effects of melatonin may vary depending on baseline sleep disturbances or other chronic comorbidities. In most included chronic MSK trials, baseline PSQI scores suggested the presence of sleep problems. However, no study analysed outcomes separately for participants with and without pre-existing sleep disturbances or other chronic conditions. This limited our ability to determine whether baseline sleep problems influenced the effects of melatonin. Fourth, subgroup analyses based on chronic MSK pain type or pain duration were not feasible because there were insufficient studies within each MSK pain subtype, and pain duration was not consistently reported. This may limit the clinical interpretability of the findings. Fifth, when multiple melatonin dose arms were available, data were extracted from the highest-dose arm, which may have introduced bias in the effect estimates. However, no clear dose–response relationship was observed in either chronic MSK pain or postoperative MSK pain analyses, suggesting that any such impact is likely limited, although it cannot be excluded.

## 5. Conclusion

In summary, melatonin seems to offer benefits for reducing pain and improving sleep in people with chronic MSK pain. These pain effects are comparable in magnitude with those of conventional analgesics (eg, NSAIDs), although the similarity in estimate magnitude does not indicate therapeutic equivalence. For people with postoperative pain, melatonin is more effective than placebo and shows similar effects to analgesic and nonanalgesic medications. Given the current evidence, melatonin may be considered as an adjunct option, with modest effects and low-to-moderate certainty. Further high-quality RCTs with standardised dosing and longer follow-up are needed to better inform melatonin's clinical use.

## Conflict of interest statement

The authors have no conflict of interest to declare.

## Supplementary Material

## Appendix A. Supplemental digital content

Supplemental digital content associated with this article can be found online at http://links.lww.com/PAIN/C537.
