# Supplementary material for: Efficacy and effectiveness of melatonin for the management of musculoskeletal pain: a systematic review and meta-analysis of placebo and active controlled trials
Source: Pain. 2026 Jun 30;167(9):2014–25. doi: 10.1097/j.pain.0000000000004045 (PMC13528901; doi:10.1097/j.pain.0000000000004045)
Supplement: Supplementary file 1 [file jop-167-2014-s001.pdf]

Supplementary appendix

Efficacy and effectiveness of melatonin for the management of musculoskeletal pain: a systematic review and meta-analysis of placebo and active controlled trials

Kangchao Wu, Ting Him Ho, Paula R Beckenkamp, et al.

**Supplementary Material S1**	Page 2
**Figure S1**	Page 16
**Figure S2**	Page 17
**Figure S3**	Page 18
**Figure S4**	Page 19
**Figure S5**	Page 20
**Figure S6**	Page 21
**Figure S7**	Page 22
**Figure S8**	Page 23
**Figure S9**	Page 24
**Figure S10**	Page 25
**Table S1**	Page 26
**Table S2**	Page 33

Supplementary Material S1. Full search strategy

Apr 10th, 2025

Sources searched:

- Embase Classic + Embase (Ovid)
- Ovid MEDLINE(R) ALL
- Cochrane Library
- Web of Science
- PsycINFO (Ovid)
- CINAHL (EBSCOhost)

Embase Classic <1947 to 1973> Part 1 of 2

Embase <1974 to 2025 April 10>

Records found: 365

1	exp musculoskeletal disease/	3141892
2	(musculoskeletal adj3 (pain or ache* or discomfort* or injur* or condition* or symptom*)).ti,ab,kw.	31703
3	exp peripheral neuropathy/	101650
4	((femoral* or median* or peroneal* or radial* or sciatica* or tibial* or ulna*) adj3 neuropath*).ti,ab,kw.	4550
5	mononeuropath*.ti,ab,kw.	3000
6	exp fibromyalgia/	28499
7	((fibromyalgia* or fibromyositis* or muscular) adj2 rheumatism*).ti,ab,kw.	108
8	exp neck pain/	34999

9 ((neck* adj3 (pain* or discomfort* or ache* or strain*)) or (cervical* adj3 (pain* or discomfort* or ache* or strain*)) or cervicalgia*).ti,ab,kw. 31797

10 exp backache/ 152849

11 (low back pain* or lumbar pain* or dorsalgia* or Lumbago* or Lumbar spine pain* or backache*).ti,ab,kw. 61003

12 exp osteoarthritis/ 184466

13 Osteoarth*.ti,ab,kw. 151069

14 exp rheumatoid arthritis/ 269880

15 (Caplan Syndrome* or Felty Syndrome* or Rheumatoid Nodule* or Rheumatoid Vasculitis* or Sjogren's Syndrome* or Still's Disease* or Rheumatoid arthrit*).ti,ab,kw. 229698

16 exp shoulder pain/ 23834

17 (Shoulder ache* or Shoulder discomfort* or Shoulder soreness* or Shoulder joint pain* or Shoulder muscle pain* or Shoulder tendonitis* or Rotator cuff pain* or Frozen shoulder* or Shoulder impingement* or Shoulder bursitis* or Shoulder arthritis* or Shoulder strain*).ti,ab,kw. 4101

18 exp pelvic pain/ 27522

19 (pelvic* adj3 (pain* or "girdle pain*" or ache* or cramp*)).ti,ab,kw. 23798

20 exp thorax pain/ 135974

21 (Thoracic pain* or Thoracic discomfort* or Thoracic soreness* or Thoracic ache* or Upper back pain* or Mid-back pain* or Thoracic spine pain* or Thoracic region pain* or Thoracic vertebrae pain* or Thoracic musculoskeletal pain* or Thoracic spine discomfort* or Thoracic spine ache* or Thoracic spine soreness*).ti,ab,kw. 3367

22	exp melatonin/	47273
23	(melatonin* or Circadin* or Slenyto* or Melatonex* or Melatonorm* or Mela-D*).ti,ab,kw.	40979
24	N-acetyl-5-methoxytryptamin.ti,ab,kw.	3
25	1 or 2 or 3 or 4 or 5 or 6 or 7 or 8 or 9 or 10 or 11 or 12 or 13 or 14 or 15 or 16 or 17 or 18 or 19 or 20 or 21	3405754
26	22 or 23 or 24	52375
27	25 and 26	3167
28	(Randomized controlled trial/ or Controlled clinical study/ or random*.ti,ab. or randomization/ or intermethod comparison/ or placebo.ti,ab. or (compare or compared or comparison).ti. or ((evaluated or evaluate or evaluating or assessed or assess) and (compare or compared or comparing or comparison)).ab. or (open adj label).ti,ab. or ((double or single or doubly or singly) adj (blind or blinded or blindly)).ti,ab. or double blind procedure/ or parallel group*1.ti,ab. or (crossover or cross over).ti,ab. or ((assign* or match or matched or allocation) adj5 (alternate or group*1 or intervention*1 or patient*1 or subject*1 or participant*1)).ti,ab. or (assigned or allocated).ti,ab. or (controlled adj7 (study or design or trial)).ti,ab. or (volunteer or volunteers).ti,ab. or human experiment/ or trial.ti.) not (((random* adj sampl* adj7 ("cross section*" or questionnaire*1 or survey* or database*1)).ti,ab. not (comparative study/ or controlled study/ or randomi?ed controlled.ti,ab. or randomly assigned.ti,ab.)) or (Cross-sectional study/ not (randomized controlled trial/ or controlled clinical study/ or controlled study/ or randomi?ed controlled.ti,ab. or control group*1.ti,ab.)) or (((case adj control*) and random*) not randomi?ed controlled).ti,ab. or (Systematic review not (trial or study)).ti. or (nonrandom* not random*).ti,ab. or "Random field*".ti,ab. or (random cluster adj3 sampl*).ti,ab. or ((review.ab. and review.pt.) not trial.ti.) or ("we searched".ab. and (review.ti. or review.pt.)) or "update review".ab. or (databases adj4 searched).ab. or ((rat or rats or mouse or mice or swine or porcine or murine or sheep or lambs or pigs or piglets or rabbit or rabbits or cat or cats or dog or dogs or cattle or bovine or monkey or monkeys or trout or marmoset*1).ti. and animal experiment/) or (Animal experiment/ not (human experiment/ or human/)))	6140616
29	27 and 28	365

Ovid MEDLINE(R) ALL <1946 to April 10, 2025>

Records found: 140

1	exp musculoskeletal disease/	1269322
2	exp musculoskeletal pain/	8405
3	1 or 2	1269535
4	(musculoskeletal adj3 (pain or ache* or discomfort* or injur* or condition* or symptom*)).ti,ab,kw.	23381
5	exp Peripheral Nervous System Diseases/	174683
6	((femoral* or median* or peroneal* or radial* or sciatica* or tibial* or ulna*) adj3 neuropath*).ti,ab,kw.	3280
7	mononeuropath*.ti,ab,kw.	1983
8	exp Fibromyalgia/	10676
9	((fibromyalgia* or fibromyositis* or muscular) adj2 rheumatism*).ti,ab,kw.	82
10	exp Neck Pain/	9306
11	((neck* adj3 (pain* or discomfort* or ache* or strain*)) or (cervical* adj3 (pain* or discomfort* or ache* or strain*)) or cervicalgia*).ti,ab,kw.	22106
12	exp Back Pain/	47740
13	(low back pain* or lumbar pain* or dorsalgia* or Lumbago* or Lumbar spine pain* or backache*).ti,ab,kw.	45478

14	exp Osteoarthritis/	84495
15	Osteoarth*.ti,ab,kw.	107879
16	exp Arthritis, Rheumatoid/	131926
17	(Caplan Syndrome* or Felty Syndrome* or Rheumatoid Nodule* or Rheumatoid Vasculitis* or Sjogren's Syndrome* or Still's Disease* or Rheumatoid arthrit*).ti,ab,kw.	147765
18	exp Shoulder Pain/	6382
19	(Shoulder ache* or Shoulder discomfort* or Shoulder soreness* or Shoulder joint pain* or Shoulder muscle pain* or Shoulder tendonitis* or Rotator cuff pain* or Frozen shoulder* or Shoulder impingement* or Shoulder bursitis* or Shoulder arthritis* or Shoulder strain*).ti,ab,kw.	3112
20	exp Pelvic Pain/	12085
21	(pelvic* adj3 (pain* or "girdle pain*" or ache* or cramp*)).ti,ab,kw.	14327
22	(Thoracic pain* or Thoracic discomfort* or Thoracic soreness* or Thoracic ache* or Upper back pain* or Mid-back pain* or Thoracic spine pain* or Thoracic region pain* or Thoracic vertebrae pain* or Thoracic musculoskeletal pain* or Thoracic spine discomfort* or Thoracic spine ache* or Thoracic spine soreness*).ti,ab,kw.	2009
23	exp Melatonin/	24873
24	(melatonin* or Circadin* or Slenyto* or Melatonex* or Melatonorm* or Mela-D*).ti,ab,kw.	32832
25	N-acetyl-5-methoxytryptamin.ti,ab,kw.	2
26	1 or 2 or 3 or 4 or 5 or 6 or 7 or 8 or 9 or 10 or 11 or 12 or 13 or 14 or 15 or 16 or 17 or 18 or 19 or 20 or 21 or 22	1578709

#	Search	Hits
27	23 or 24 or 25	34588
28	26 and 27	701
29	(Randomized controlled trial/ or Controlled clinical study/ or random*.ti,ab. or randomization/ or intermethod comparison/ or placebo.ti,ab. or (compare or compared or comparison).ti. or ((evaluated or evaluate or evaluating or assessed or assess) and (compare or compared or comparing or comparison)).ab. or (open adj label).ti,ab. or ((double or single or doubly or singly) adj (blind or blinded or blindly)).ti,ab. or double blind procedure/ or parallel group*1.ti,ab. or (crossover or cross over).ti,ab. or ((assign* or match or matched or allocation) adj5 (alternate or group*1 or intervention*1 or patient*1 or subject*1 or participant*1)).ti,ab. or (assigned or allocated).ti,ab. or (controlled adj7 (study or design or trial)).ti,ab. or (volunteer or volunteers).ti,ab. or human experiment/ or trial.ti.) not (((random* adj sampl* adj7 ("cross section*" or questionnaire*1 or survey* or database*1)).ti,ab. not (comparative study/ or controlled study/ or randomi?ed controlled.ti,ab. or randomly assigned.ti,ab.)) or (Cross-sectional study/ not (randomized controlled trial/ or controlled clinical study/ or controlled study/ or randomi?ed controlled.ti,ab. or control group*1.ti,ab.)) or (((case adj control*) and random*) not randomi?ed controlled).ti,ab. or (Systematic review not (trial or study)).ti. or (nonrandom* not random*).ti,ab. or "Random field*".ti,ab. or (random cluster adj3 sampl*).ti,ab. or ((review.ab. and review.pt.) not trial.ti.) or ("we searched".ab. and (review.ti. or review.pt.)) or "update review".ab. or (databases adj4 searched).ab. or ((rat or rats or mouse or mice or swine or porcine or murine or sheep or lambs or pigs or piglets or rabbit or rabbits or cat or cats or dog or dogs or cattle or bovine or monkey or monkeys or trout or marmoset*1).ti. and animal experiment/) or (Animal experiment/ not (human experiment/ or human/)))	4053601
30	28 and 29	140

Cochrane Library

Date Run: 10/04/2025

Records found: 137

ID	Search	Hits
#1	MeSH descriptor: [Musculoskeletal Pain] explode all trees	1723

#2 (Musculoskeletal NEXT (Pain* or ache* or discomfort* or injur* or condition* or symptom*)):ti,ab,kw (Word variations have been searched) in Trials 4789

#3 MeSH descriptor: [Peripheral Nervous System Diseases] explode all trees 8417

#4 ((Femoral* or Median* or Peroneal* or Radial* or Sciatica* or Tibial* or Ulna*) NEXT neuropath*):ti,ab,kw (Word variations have been searched) in Trials 158

#5 (mononeuropath*):ti,ab,kw (Word variations have been searched) in Trials 100

#6 MeSH descriptor: [Fibromyalgia] explode all trees 2051

#7 (Fibromyalgia* or fibromyositis* or muscular NEXT rheumatism*):ti,ab,kw (Word variations have been searched) in Trials 3991

#8 MeSH descriptor: [Neck Pain] explode all trees 2212

#9 (Neck* NEXT (Pain* or Discomfort* or Ache* or Strain*)):ti,ab,kw OR (Cervical* NEXT (Pain* or Discomfort* or Ache* or Strain*)):ti,ab,kw OR (Cervicalgia*):ti,ab,kw (Word variations have been searched) in Trials 5935

#10 MeSH descriptor: [Back Pain] explode all trees 7743

#11 (low back pain* or lumbar pain* or dorsalgia* or Lumbago* or Lumbar spine pain* or backache*):ti,ab,kw (Word variations have been searched) in Trials 26092

#12 MeSH descriptor: [Osteoarthritis] explode all trees 11122

#13 (Osteoarth*):ti,ab,kw (Word variations have been searched) in Trials 25936

#14 MeSH descriptor: [Arthritis, Rheumatoid] explode all trees 8054

#15 (Caplan Syndrome* or Felty Syndrome* or Rheumatoid Nodule* or Rheumatoid Vasculitis* or Sjogren's Syndrome* or Still's Disease* or Rheumatoid arthrit*):ti,ab,kw (Word variations have been searched) in Trials 38714

#16 MeSH descriptor: [Shoulder Pain] explode all trees 1529

#17 (Shoulder ache* or Shoulder discomfort* or Shoulder soreness* or Shoulder joint pain* or Shoulder muscle pain* or Shoulder tendonitis* or Rotator cuff pain* or Frozen shoulder* or Shoulder impingement* or Shoulder bursitis* or Shoulder arthritis* or Shoulder strain*):ti,ab,kw (Word variations have been searched) in Trials 8218

#18 MeSH descriptor: [Pelvic Pain] explode all trees 1845

#19 (Pelvic* NEAR/3 (pain* or girdle pain* or ache* or cramp*)):ti,ab,kw (Word variations have been searched) in Trials 3549

#20 (Thoracic pain* or Thoracic discomfort* or Thoracic soreness* or Thoracic ache* or Upper back pain* or Mid-back pain* or Thoracic spine pain* or Thoracic region pain* or Thoracic vertebrae pain* or Thoracic musculoskeletal pain* or Thoracic spine discomfort* or Thoracic spine ache* or Thoracic spine soreness*):ti,ab,kw (Word variations have been searched) in Trials 7334

#21 MeSH descriptor: [Melatonin] explode all trees 1757

#22 (melatonin* or Circadin* or Slenyto* or Melatonex* or Melatonorm* or Mela-D*):ti,ab,kw OR ("N-acetyl-5-methoxytryptamin"):ti,ab,kw (Word variations have been searched) in Trials 4281

#23 #21 or #22 in Trials 4281

#24 #1 or #2 or #3 or #4 or #5 or #6 or #7 or #8 or #9 or #10 or #11 or #12 or #13 or #14 or #15 or #16 or #17 or #18 or #19 or #20 123662

#25 #23 and #24 137

Web of Science Search Strategy

Database: Web of Science Core Collection

Records found: 119

Searches:

1: TS=((“Musculoskeletal*” NEAR/3 (“pain*” or “ach*” or “discomfort*” or “injur*” or “condition*” or “symptom*”))) Date Run: Thu Apr 10 2025 16:13:37 GMT+1000 Results: 34143

2: TS=(“Femoral Neuropath*” or “Median Neuropath*” or “Peroneal Neuropath*” or “Radial Neuropath*” or “Sciatic Neuropath*” or “Tibial Neuropath*” or “Ulnar Neuropath” or “Mononeuropath*”) Date Run: Thu Apr 10 2025 16:14:15 GMT+1000 Results: 4345

3: TS=("Fibromyalgia*" or "muscular rheumatism" or "fibrositi*" or "diffuse myofascial pain syndrome*" or "Fibromyositis*") Date Run: Thu Apr 10 2025 16:14:39 GMT+1000 Results: 24117

4: TS=(“Neck pain*” or “Cervical pain*” or “Cervicalgia*” or “cervical discomfort*” or “Neck ache*” or “Cervical soreness*” or “Neck strain*” or “Cervical discomfort*” or “Cervical ache*” or "neck-ache*") Date Run: Thu Apr 10 2025 16:15:22 GMT+1000 Results: 18983

5: TS=(“back pain*” or “low back pain*” or “lumbar pain*” or “lumbago*” or “dorsalgia*” or “spinal pain*” or “vertebral pain*” or “backache*” or “lumbar spine pain*” or "back-ache*") Date Run: Thu Apr 10 2025 16:15:39 GMT+1000 Results: 101938

6: TS=("osteoarth*") Date Run: Thu Apr 10 2025 16:15:59 GMT+1000 Results: 150720

7: TS=(“RA” or "Rheumatoid* arthrit*" or “Rheumatoid disease*” or “Inflammatory arthritis*” or “Chronic autoimmune arthritis*” or “Rheumatoid synovitis*”) Date Run: Thu Apr 10 2025 16:16:20 GMT+1000

Results: 322677

8: TS=(“Shoulder ache*” or “Shoulder discomfort*” or “Shoulder soreness*” or “Shoulder joint pain*” or “Shoulder muscle pain*” or “Shoulder tendonitis*” or “Rotator cuff pain*” or “Frozen shoulder*” or “Shoulder impingement*” or “Shoulder bursitis*” or “Shoulder arthritis*” or “Shoulder strain*”) Date Run: Thu Apr 10 2025 16:16:39 GMT+1000

Results: 4185

9: TS= ("Pelvic*" NEAR/3 ("pain*" or "girdle pain*" or "ache*" or "cramp*")) Date Run: Thu Apr 10 2025 16:17:07 GMT+1000

Results: 18362

10: TS=("Knee pain*" or "Knee discomfort*" or "Knee soreness*" or "Knee ache*" or "Knee swelling*" or "Knee joint pain*" or "Knee arthrit*" or "Knee ligament pain*" or "Knee tendon pain*" or "Knee meniscus pain*" or "Knee osteoarth*" or "Knee bursitis*" or "patellofemoral pain*" or "knee tedin*" or "patellar tedin*" or "jump* knee*" or "run* knee*" or "menisc* tear*") Date Run: Thu Apr 10 2025 16:17:23 GMT+1000

Results: 53722

11: TS=(“Thoracic pain*” or “Thoracic discomfort*” or “Thoracic soreness*” or “Thoracic ache*” or “Upper back pain*” or “Mid-back pain*” or “Thoracic spine pain*” or “Thoracic region pain*” or “Thoracic vertebrae pain*” or “Thoracic musculoskeletal pain*” or “Thoracic spine discomfort*” or “Thoracic spine ache*” or “Thoracic spine soreness*”)

Date Run: Thu Apr 10 2025 16:17:38 GMT+1000

Results: 1715

12: #11 OR #10 OR #9 OR #8 OR #7 OR #6 OR #5 OR #4 OR #3 OR #2 OR #1 Date Run: Thu Apr 10 2025 16:18:04 GMT+1000

Results: 642842

13: TS=("melatonin*" or "Circadin*" or "Slenyto*" or "Melatonex*" or "Adaflex*" or "Ceyesto*" or "Syncrodin*" or "Melatonorm*" or "Mela-D*" or "N-acetyl-5-methoxytryptamine*") Date Run: Thu Apr 10 2025 16:18:23 GMT+1000

Results:
50533

14: #12 AND #13 Date Run:
Thu Apr 10 2025 16:18:46 GMT+1000

Results:

513

15: TS=("Controlled clinical trial*" or "clinical trial*" or "Trial*" or "crossover*" or "cross over*" or "random" or "placebo" or ("singl*" or "doubl*" or "trebl*" or "tripl*") Near/1 ("blind*" or "mask*") or "systematic review*" or "randomised controlled trial*" or "random assignment*" or "control groups*" or "cross-over studies*") Date Run:
Thu Apr 10 2025 16:19:10 GMT+1000

Results:

4030001

16: #14 AND #15 Date Run:
Thu Apr 10 2025 16:19:30 GMT+1000

Results:

119

APA PsycInfo <1806 to March 2025 Week 5>

Records found: 12

1 exp
Musculoskeletal Disorders/ 22476

2

(musculos
keletal adj3 (pain or ache* or discomfort* or injur* or condition* or symptom*)).ti,ab.
3803

3 exp
Peripheral Neuropathy/ 3693

4 ((femoral*
or median* or peroneal* or radial* or sciatica* or tibial* or ulna*) adj3 neuropath*).ti,ab.
139

5

mononeur
opath*.ti,ab. 233

6 exp
Fibromyalgia/ 2633

7	((fibromyalgia* or fibromyositis* or muscular) adj2 rheumatism*).ti,ab.	10
8	Neck Pain.mp.	1608
9	((neck* adj3 (pain* or discomfort* or ache* or strain*)) or (cervical* adj3 (pain* or discomfort* or ache* or strain*)) or cervicalgia*).ti,ab.	1922
10	exp Back Pain/	5076
11	(low back pain* or lumbar pain* or dorsalgia* or Lumbago* or Lumbar spine pain* or backache*).ti,ab.	4698
12	exp arthritis/	5154
13	Osteoarth*.ti,ab.	2551
14	exp Rheumatoid Arthritis/	2218
15	(Caplan Syndrome* or Felty Syndrome* or Rheumatoid Nodule* or Rheumatoid Vasculitis* or Sjogren's Syndrome* or Still's Disease* or Rheumatoid arthrit*).ti,ab.	3123
16	Shoulder Pain.mp.	724
17	(Shoulder ache* or Shoulder discomfort* or Shoulder soreness* or Shoulder joint pain* or Shoulder muscle pain* or Shoulder tendonitis* or Rotator cuff pain* or Frozen shoulder* or Shoulder impingement* or Shoulder bursitis* or Shoulder arthritis* or Shoulder strain*).ti,ab.	96
18	Pelvic Pain.mp.	919
19	(pelvic* adj3 (pain* or "girdle pain*" or ache* or cramp*)).ti,ab.	851
20	thorax pain.mp.	1

21 (Thoracic pain* or Thoracic discomfort* or Thoracic soreness* or Thoracic ache* or Upper back pain* or Mid-back pain* or Thoracic spine pain* or Thoracic region pain* or Thoracic vertebrae pain* or Thoracic musculoskeletal pain* or Thoracic spine discomfort* or Thoracic spine ache* or Thoracic spine soreness*).ti,ab. 88

22 (Knee pain* or Knee discomfort* or Knee soreness* or Knee ache* or Knee swelling* or Knee joint pain* or Knee arthrit* or Knee ligament pain* or Knee tendon pain* or Knee meniscus pain* or Knee osteoarth* or Knee bursitis* or patellofemoral pain* or knee tedin* or patellar tedin* or jump* knee* or run* knee* or menisc* tear*).ti,ab. 1058

23 1 or 2 or 3 or 4 or 5 or 6 or 7 or 8 or 9 or 10 or 11 or 12 or 13 or 14 or 15 or 16 or 17 or 18 or 19 or 20 or 21 or 22 38471

24 exp Melatonin/ 4014

25 (melatonin* or Circadin* or Slenyto* or Melatonex* or Melatonorm* or Mela-D*).ti,ab. 5431

26 24 or 25 5580

27 23 and 26 58

28 (Randomized controlled trial/ or Controlled clinical study/ or random*.ti,ab. or randomization/ or intermethod comparison/ or placebo.ti,ab. or (compare or compared or comparison).ti. or ((evaluated or evaluate or evaluating or assessed or assess) and (compare or compared or comparing or comparison)).ab. or (open adj label).ti,ab. or ((double or single or doubly or singly) adj (blind or blinded or blindly)).ti,ab. or double blind procedure/ or parallel group*1.ti,ab. or (crossover or cross over).ti,ab. or ((assign* or match or matched or allocation) adj5 (alternate or group*1 or intervention*1 or patient*1 or subject*1 or participant*1)).ti,ab. or (assigned or allocated).ti,ab. or (controlled adj7 (study or design or trial)).ti,ab. or (volunteer or volunteers).ti,ab. or human experiment/ or trial.ti.) not (((random* adj sampl* adj7 ("cross section*" or questionnaire*1 or survey* or database*1)).ti,ab. not (comparative study/ or controlled study/ or randomi?ed controlled.ti,ab. or randomly assigned.ti,ab.)) or (Cross-sectional study/ not (randomized controlled trial/ or controlled clinical study/ or controlled study/ or randomi?ed controlled.ti,ab. or control group*1.ti,ab.)) or (((case adj control*) and random*) not randomi?ed controlled).ti,ab. or (Systematic review not (trial or study)).ti. or (nonrandom* not random*).ti,ab. or "Random field*".ti,ab. or (random cluster adj3 sampl*).ti,ab. or

	((review.ab. and review.pt.) not trial.ti.) or ("we searched".ab. and (review.ti. or review.pt.)) or "update review".ab. or (databases adj4 searched).ab. or ((rat or rats or mouse or mice or swine or porcine or murine or sheep or lambs or pigs or piglets or rabbit or rabbits or cat or cats or dog or dogs or cattle or bovine or monkey or monkeys or trout or marmoset*1).ti. and animal experiment/) or (Animal experiment/ not (human experiment/ or human/)))	623908
29	27 and 28	12

Figure S1. Risk of bias assessment by individual study

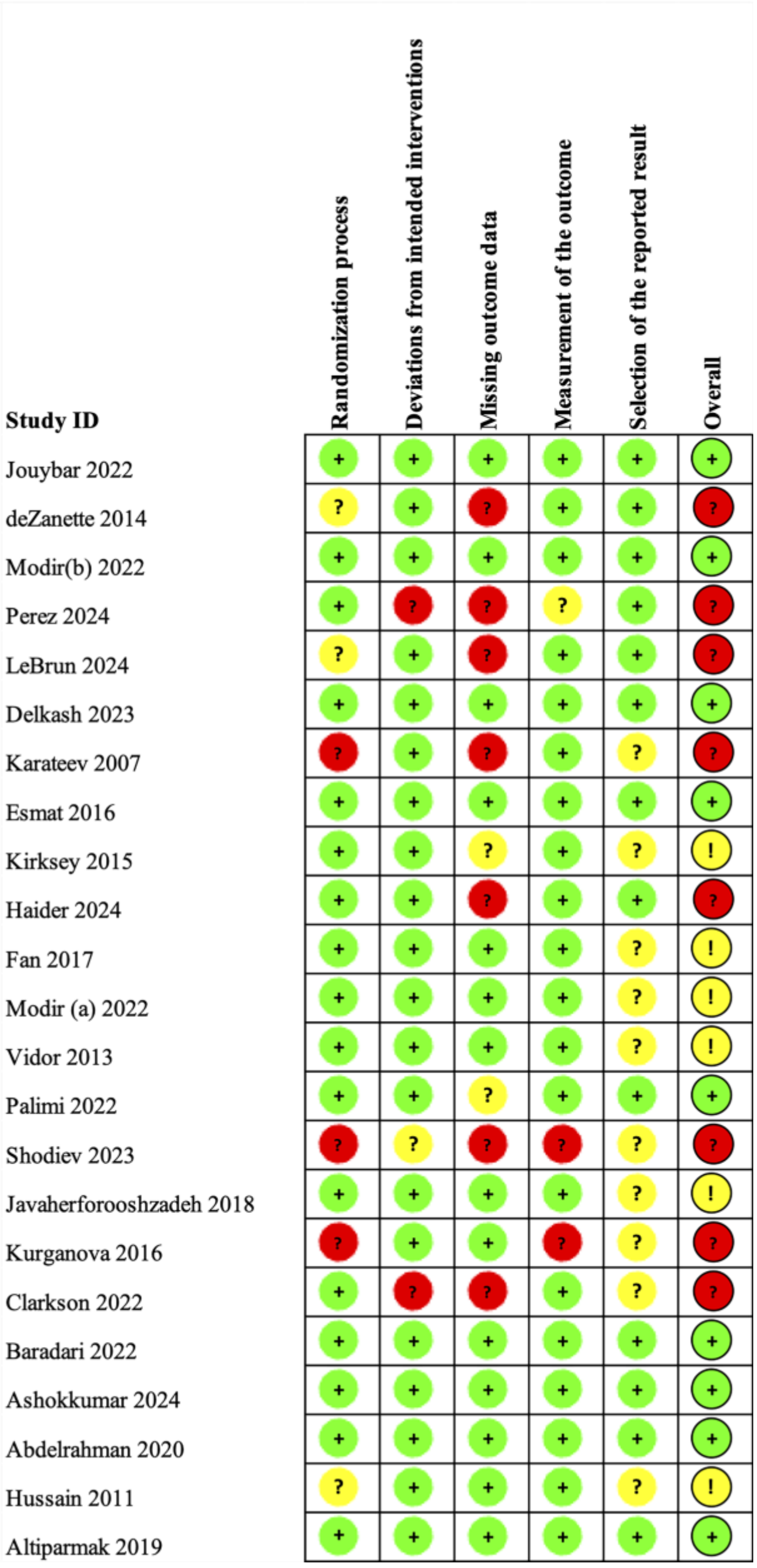

Figure S2. Risk of bias assessment by domain

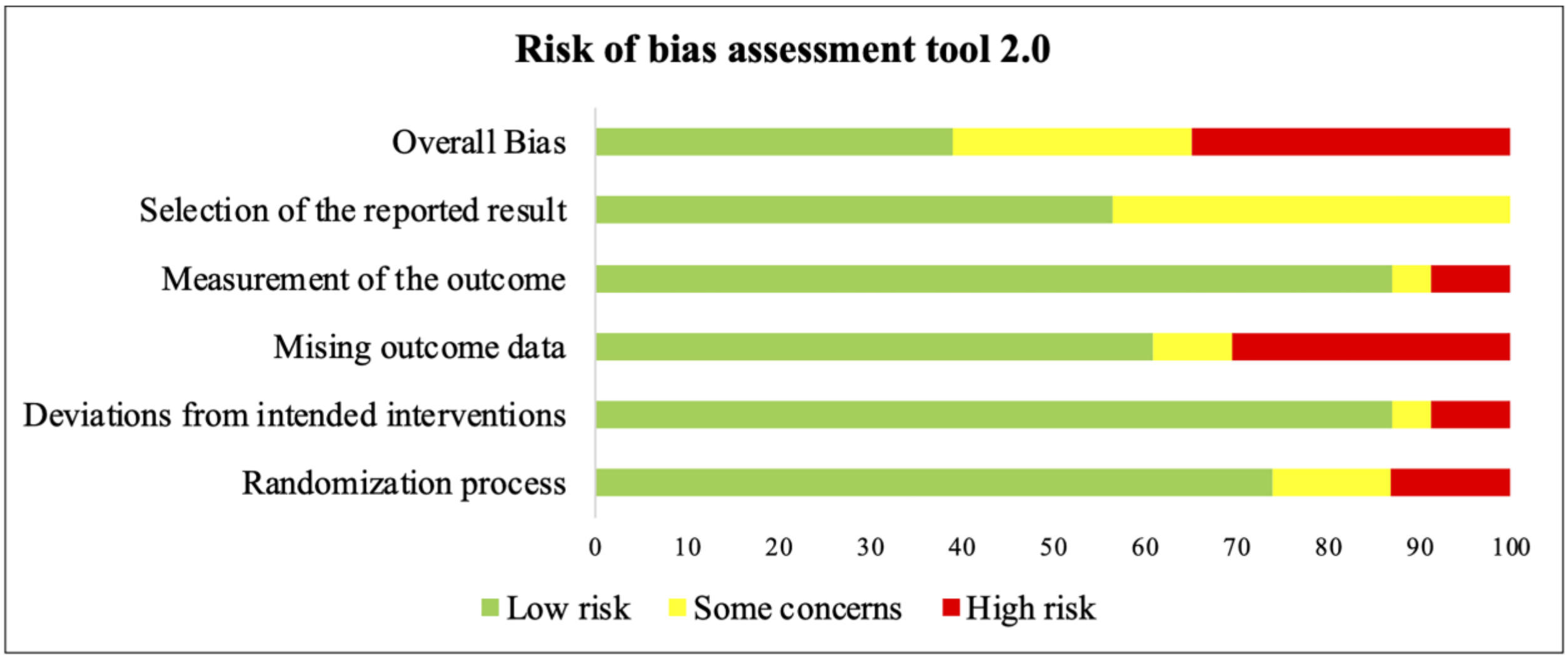

Figure S3. Subgroup analyses by type of surgical condition

Study or Subgroup	Melatonin Mean	Melatonin SD	Melatonin Total	Control Mean	Control SD	Control Total	Weight	Mean Difference IV, Random, 95% CI
**19.1.1 fracture-related surgeries**								
Abdelrahman 2020	15	7.4	40	20	0.01	40	8.2%	-5.00 [-7.29, -2.71]
Ashokkumar 2024	8.4	3.7	32	11.2	3.3	32	8.7%	-2.80 [-4.52, -1.08]
Modir (b) 2022	54.7	5.7	30	55.7	5	30	7.9%	-1.00 [-3.71, 1.71]
Modir (b) 2022	54.7	5.7	30	52.3	4.3	30	8.0%	2.40 [-0.15, 4.95]
Modir (b) 2022	54.7	5.7	30	52.3	6.3	30	7.6%	2.40 [-0.64, 5.44]
**Subtotal (95% CI)**			**162**			**162**	**40.4%**	**-0.91 [-3.67, 1.85]**
Heterogeneity: Tau² = 8.32; Chi² = 26.55, df = 4 (P < 0.0001); I² = 85%								
Test for overall effect: Z = 0.65 (P = 0.52)								
**19.1.2 spine surgeries**								
Baradari 2022	19	24	20	31.5	25	20	1.3%	-12.50 [-27.69, 2.69]
Esmat 2016	32.8	4.5	25	38	6.9	25	7.4%	-5.20 [-8.43, -1.97]
Esmat 2016	32.8	4.5	25	32.8	4.5	25	8.1%	0.00 [-2.49, 2.49]
Javaherforooshzadeh 2018	18	5.8	30	10	3.7	30	8.1%	8.00 [5.54, 10.46]
Jouybar 2022	31.2	16.3	33	25.6	10.7	33	4.4%	5.60 [-1.05, 12.25]
Jouybar 2022	31.2	16.3	33	18.5	7.5	33	4.7%	12.70 [6.58, 18.82]
Modir (a) 2022	41.6	4.61	30	42.3	4.3	30	8.3%	-0.70 [-2.96, 1.56]
**Subtotal (95% CI)**			**196**			**196**	**42.2%**	**2.08 [-2.46, 6.63]**
Heterogeneity: Tau² = 30.00; Chi² = 65.90, df = 6 (P < 0.00001); I² = 91%								
Test for overall effect: Z = 0.90 (P = 0.37)								
**19.1.3 joint replacement surgeries**								
Fan 2017	31	2	69	32	2	70	9.3%	-1.00 [-1.66, -0.34]
Haider 2024	46	24	64	42	24	64	3.3%	4.00 [-4.32, 12.32]
Kirksey 2015	20.526	27.381	19	24.211	24.566	19	1.2%	-3.68 [-20.23, 12.86]
LeBrun 2024	36.2	23.8	73	35.6	23	67	3.7%	0.60 [-7.15, 8.35]
**Subtotal (95% CI)**			**225**			**220**	**17.4%**	**-0.96 [-1.62, -0.30]**
Heterogeneity: Tau² = 0.00; Chi² = 1.64, df = 3 (P = 0.65); I² = 0%								
Test for overall effect: Z = 2.85 (P = 0.004)								
**Total (95% CI)**			**583**			**578**	**100.0%**	**0.54 [-1.35, 2.44]**
Heterogeneity: Tau² = 9.99; Chi² = 111.05, df = 15 (P < 0.00001); I² = 86%								
Test for overall effect: Z = 0.56 (P = 0.57)								
Test for subgroup differences: Chi² = 1.69, df = 2 (P = 0.43), I² = 0%								

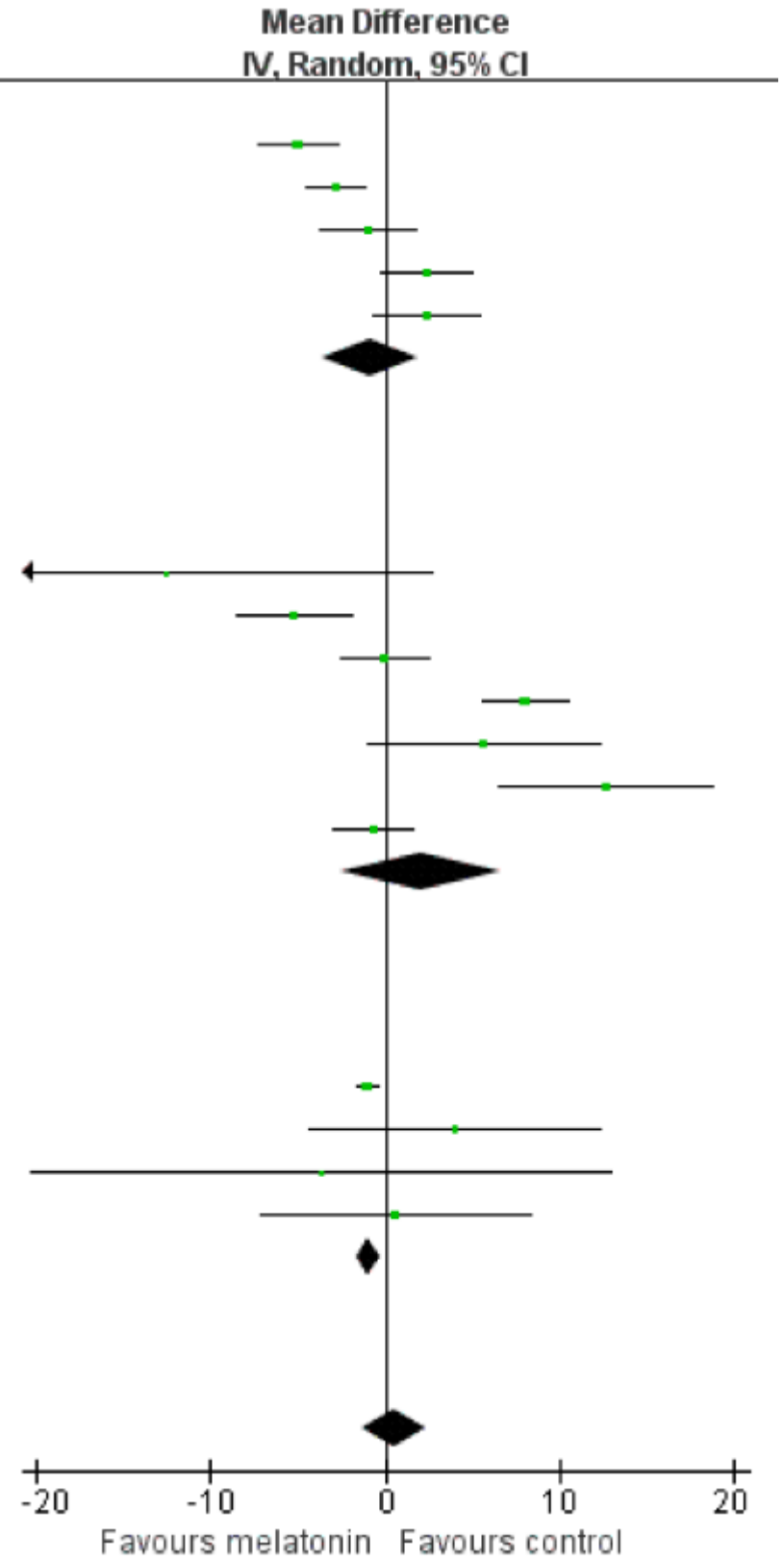

Figure S4. Effect of melatonin on sleep quality in people with chronic MSK pain

	Melatonin			Control				Mean Difference
Study or Subgroup	Mean	SD	Total	Mean	SD	Total	Weight	IV, Random, 95% CI
**1.2.1 melatonin vs placebo**								
Altiparmak 2019	15.232	2.48	40	15.71	3.62	40	17.1%	-0.48 [-1.84, 0.88]
Karateev 2007	32.49	17.08	17	40.41	23.75	18	10.7%	-7.92 [-21.57, 5.73]
Palimi 2022	19.75	11.71	32	37.03	13.76	32	15.3%	-17.28 [-23.54, -11.02]
Vidor 2013	24	12	16	56.6	16.2	16	13.1%	-32.60 [-42.48, -22.72]
**Subtotal (95% CI)**			**105**			**106**	**56.3%**	**-14.35 [-29.13, 0.43]**
Heterogeneity: Tau² = 207.67; Chi² = 65.23, df = 3 (P < 0.00001); I² = 95%								
Test for overall effect: Z = 1.90 (P = 0.06)								
**1.2.2 melatonin vs analgesic medications**								
Kurganova 2016	18.71	8.85	29	26.18	7.33	29	16.3%	-7.47 [-11.65, -3.29]
Shodiev 2023	33.32	8.81	12	42.84	7.04	12	15.2%	-9.52 [-15.90, -3.14]
**Subtotal (95% CI)**			**41**			**41**	**31.5%**	**-8.09 [-11.58, -4.59]**
Heterogeneity: Tau² = 0.00; Chi² = 0.28, df = 1 (P = 0.60); I² = 0%								
Test for overall effect: Z = 4.53 (P < 0.00001)								
**1.2.3 melatonin vs non-analgesic medications**								
Hussain 2011	56.7	18.7	27	63.6	22	24	12.2%	-6.90 [-18.18, 4.38]
**Subtotal (95% CI)**			**27**			**24**	**12.2%**	**-6.90 [-18.18, 4.38]**
Heterogeneity: Not applicable								
Test for overall effect: Z = 1.20 (P = 0.23)								
**Total (95% CI)**			**173**			**171**	**100.0%**	**-11.35 [-18.65, -4.06]**
Heterogeneity: Tau² = 80.45; Chi² = 76.76, df = 6 (P < 0.00001); I² = 92%								
Test for overall effect: Z = 3.05 (P = 0.002)								
Test for subgroup differences: Chi² = 0.72, df = 2 (P = 0.70), I² = 0%								

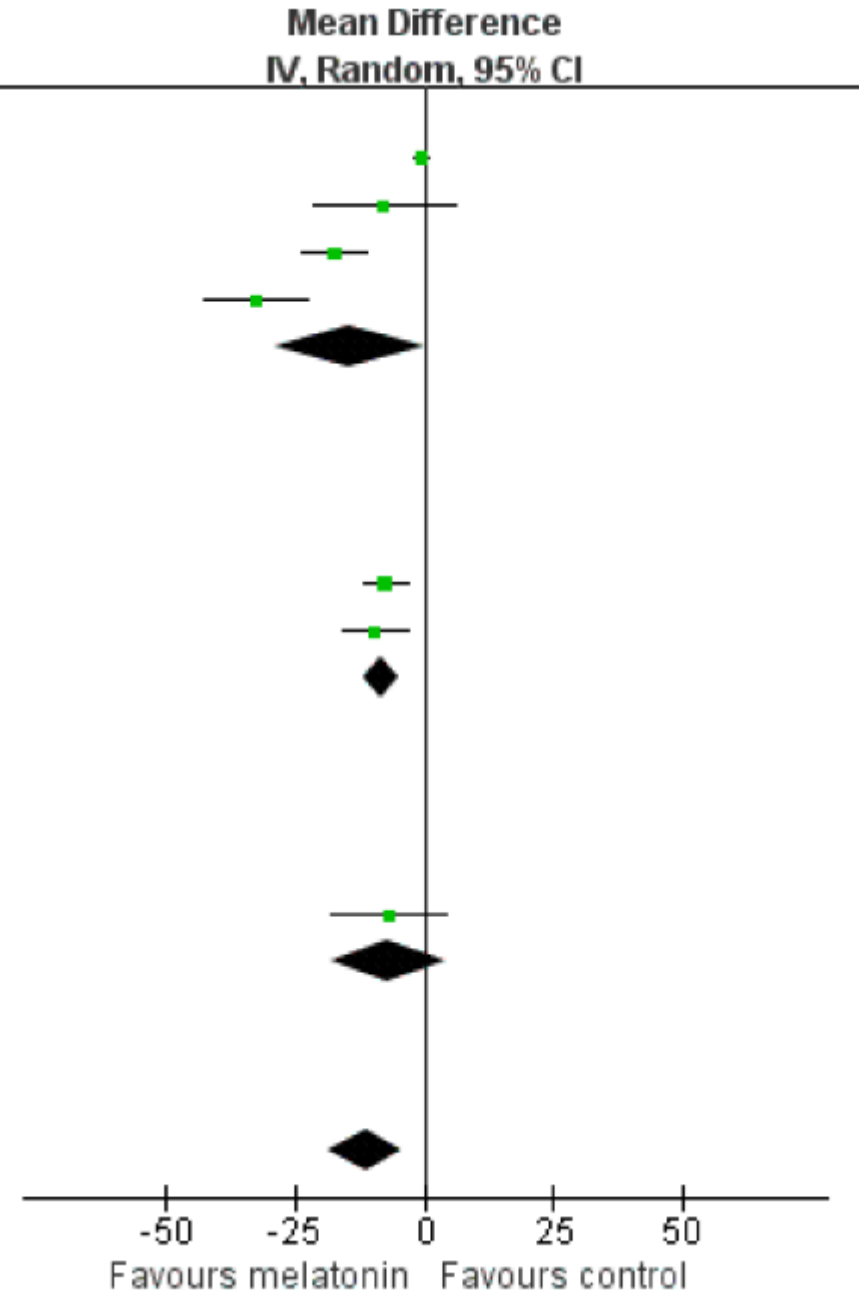

Figure S5. Effect of melatonin on sleep quality in people with postoperative MSK pain

Study or Subgroup	Melatonin Mean	Melatonin SD	Melatonin Total	Control Mean	Control SD	Control Total	Weight	Mean Difference IV, Random, 95% CI
**10.2.1 melatonin vs placebo**								
Clarkson 2022	41.89	21.9	50	41.41	22.37	53	10.4%	0.48 [-8.07, 9.03]
Fan 2017	33.44	2.34	69	40.55	3.5	70	28.9%	-7.11 [-8.10, -6.12]
Haider 2024	70.05	22.77	64	68.56	17.08	64	13.3%	1.49 [-5.48, 8.46]
Kirksey 2015	89	9.119	20	90.53	8.481	19	16.7%	-1.53 [-7.05, 3.99]
LeBrun 2024	48.22	19.94	73	49.93	21.04	67	13.6%	-1.71 [-8.51, 5.09]
**Subtotal (95% CI)**			**276**			**273**	**83.0%**	**-2.47 [-6.79, 1.84]**
Heterogeneity: Tau² = 15.54; Chi² = 14.05, df = 4 (P = 0.007); I² = 72%								
Test for overall effect: Z = 1.12 (P = 0.26)								
**10.2.2 melatonin vs sleep hygiene recommendations**								
Perez 2024	31.37	12.14	40	37.94	12.57	40	17.0%	-6.57 [-11.99, -1.15]
**Subtotal (95% CI)**			**40**			**40**	**17.0%**	**-6.57 [-11.99, -1.15]**
Heterogeneity: Not applicable								
Test for overall effect: Z = 2.38 (P = 0.02)								
**Total (95% CI)**			**316**			**313**	**100.0%**	**-3.42 [-6.84, 0.00]**
Heterogeneity: Tau² = 10.27; Chi² = 14.05, df = 5 (P = 0.02); I² = 64%								
Test for overall effect: Z = 1.96 (P = 0.05)								
Test for subgroup differences: Chi² = 1.35, df = 1 (P = 0.25), I² = 25.7%								

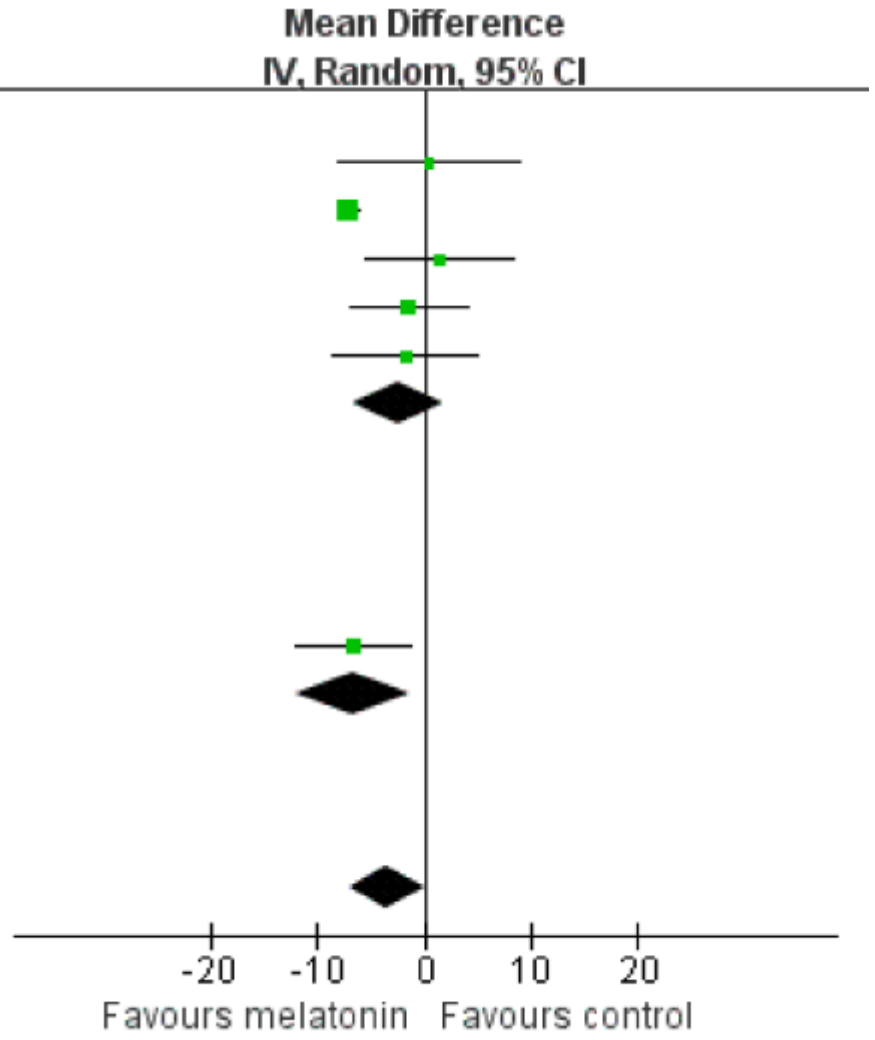

Figure S6 Studies ordered by melatonin dose (chronic MSK pain)

Study or Subgroup	Melatonin Mean	Melatonin SD	Melatonin Total	Control Mean	Control SD	Control Total	Weight	Mean Difference IV, Random, 95% CI	Dosage	Treatment Duration
deZanette 2014	47.53	21.96	21	50.02	25.6	21	5.4%	-2.49 [-16.92, 11.94]	10mg	6 weeks
Hussain 2011	60.8	18.7	27	65.5	16.7	24	9.1%	-4.70 [-14.42, 5.02]	5mg	8 weeks
Vidor 2013	21.3	18.2	16	38	20.5	16	6.0%	-16.70 [-30.13, -3.27]	5mg	4 weeks
Kurganova 2016	20	22	29	34.4	16.2	29	8.8%	-14.40 [-24.34, -4.46]	3mg	12 weeks
Kurganova 2016	9	11.2	31	22.5	16	29	12.4%	-13.50 [-20.53, -6.47]	3mg	12 weeks
Palimi 2022	44.84	24.77	32	59.21	24.59	32	6.9%	-14.37 [-26.46, -2.28]	3mg	8 weeks
Altiparmak 2019	33.3	7.3	40	38.8	9.1	40	17.7%	-5.50 [-9.12, -1.88]	3mg	4 weeks
Shodiev 2023	60.32	14.8	12	67.12	4.12	12	10.2%	-6.80 [-15.49, 1.89]	3mg	4 weeks
Karateev 2007	54.4	18	17	43.4	26.4	18	5.1%	11.00 [-3.90, 25.90]	3mg	3 weeks
Delkash 2024	26.8	5.5	28	39.65	6.5	29	18.4%	-12.85 [-15.97, -9.73]	3-10mg	12 weeks
**Total (95% CI)**			**253**			**250**	**100.0%**	**-8.96 [-12.85, -5.08]**		

Heterogeneity: Tau² = 18.79; Chi² = 22.50, df = 9 (P = 0.007); I² = 60%

Test for overall effect: Z = 4.52 (P < 0.00001)

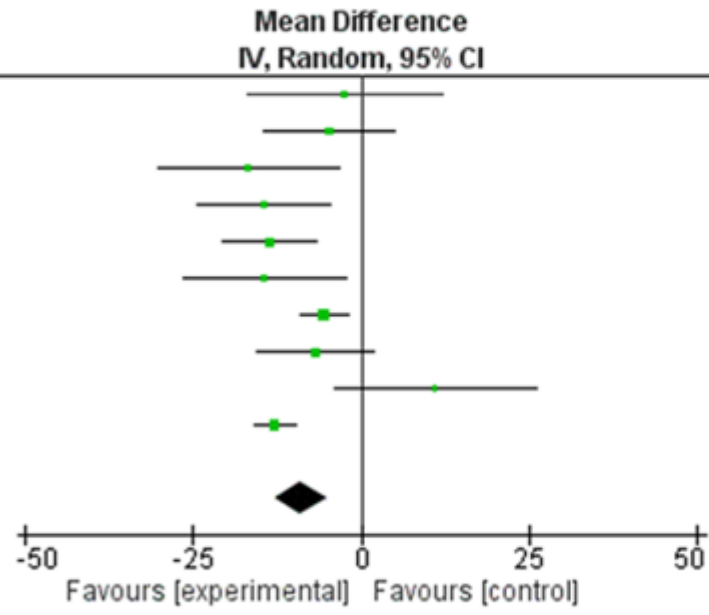

Figure S7 Studies ordered by melatonin dose (postoperative MSK pain)

	Melatonin			Control				Mean Difference		
Study or Subgroup	Mean	SD	Total	Mean	SD	Total	Weight	IV, Random, 95% CI	Dosage	Number of Administration
Ashokkumar 2024	8.4	3.7	32	11.2	3.3	32	8.7%	-2.80 [-4.52, -1.08]	10mg	15 times
Jouybar 2022	31.2	16.3	33	18.5	7.5	33	4.7%	12.70 [6.58, 18.82]	10mg	single dose
Jouybar 2022	31.2	16.3	33	25.6	10.7	33	4.4%	5.60 [-1.05, 12.25]	10mg	single dose
Baradari 2022	19	24	20	31.5	25	20	1.3%	-12.50 [-27.69, 2.69]	10mg	single dose
Esmat 2016	32.8	4.5	25	38	6.9	25	7.4%	-5.20 [-8.43, -1.97]	7mg	single dose
Esmat 2016	32.8	4.5	25	32.8	4.5	25	8.1%	0.00 [-2.49, 2.49]	7mg	single dose
Javaherforooshzadeh 2018	18	5.8	30	10	3.7	30	8.1%	8.00 [5.54, 10.46]	6mg	single dose
Modir (a) 2022	41.6	4.61	30	42.3	4.3	30	8.3%	-0.70 [-2.96, 1.56]	6mg	single dose
Modir (b) 2022	54.7	5.7	30	52.3	4.3	30	8.0%	2.40 [-0.15, 4.95]	6mg	single dose
Modir (b) 2022	54.7	5.7	30	55.7	5	30	7.9%	-1.00 [-3.71, 1.71]	6mg	single dose
Modir (b) 2022	54.7	5.7	30	52.3	6.3	30	7.6%	2.40 [-0.64, 5.44]	6mg	single dose
LeBrun 2024	36.2	23.8	73	35.6	23	67	3.7%	0.60 [-7.15, 8.35]	5mg	42 times
Haider 2024	46	24	64	42	24	64	3.3%	4.00 [-4.32, 12.32]	5mg	14 times
Kirksey 2015	20.526	27.381	19	24.211	24.566	19	1.2%	-3.68 [-20.23, 12.86]	5mg	7 times
Abdelrahman 2020	15	7.4	40	20	0.01	40	8.2%	-5.00 [-7.29, -2.71]	5mg	4 times
Fan 2017	31	2	69	32	2	70	9.3%	-1.00 [-1.66, -0.34]	1mg	7 times
**Total (95% CI)**			**583**			**578**	**100.0%**	**0.54 [-1.35, 2.44]**		

Heterogeneity: Tau² = 9.99; Chi² = 111.05, df = 15 (P < 0.00001); I² = 86%

Test for overall effect: Z = 0.56 (P = 0.57)

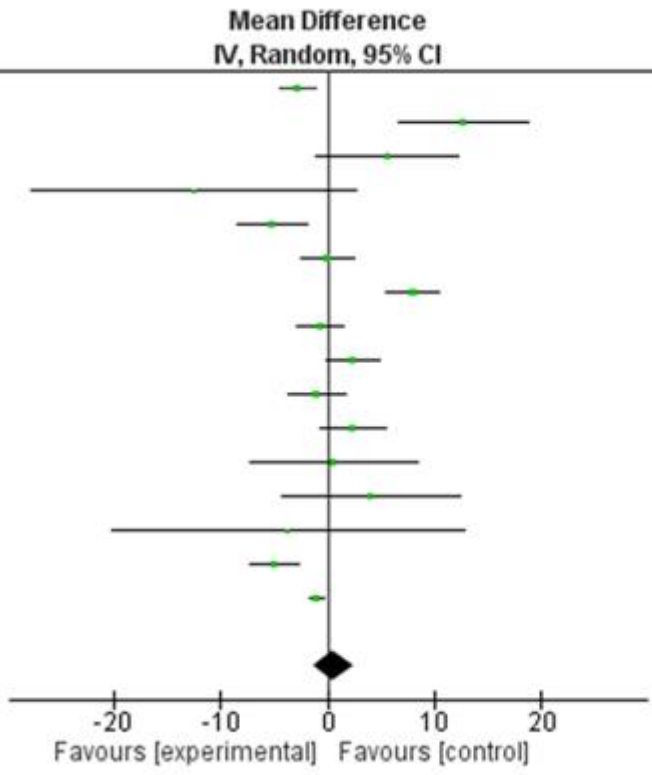

Figure S8 Studies ordered by treatment duration (chronic MSK pain)

	Melatonin			Control				Mean Difference	Treatment	
Study or Subgroup	Mean	SD	Total	Mean	SD	Total	Weight	IV, Random, 95% CI	Duration	Dosage
Delkash 2024	26.8	5.5	28	39.65	6.5	29	18.4%	-12.85 [-15.97, -9.73]	12 weeks	3-10mg
Kurganova 2016	20	22	29	34.4	16.2	29	8.8%	-14.40 [-24.34, -4.46]	12 weeks	3mg
Kurganova 2016	9	11.2	31	22.5	16	29	12.4%	-13.50 [-20.53, -6.47]	12 weeks	3mg
Palimi 2022	44.84	24.77	32	59.21	24.59	32	6.9%	-14.37 [-26.46, -2.28]	8 weeks	3mg
Hussain 2011	60.8	18.7	27	65.5	16.7	24	9.1%	-4.70 [-14.42, 5.02]	8 weeks	5mg
deZanette 2014	47.53	21.96	21	50.02	25.6	21	5.4%	-2.49 [-16.92, 11.94]	6 weeks	10mg
Shodiev 2023	60.32	14.8	12	67.12	4.12	12	10.2%	-6.80 [-15.49, 1.89]	4 weeks	3mg
Altiparmak 2019	33.3	7.3	40	38.8	9.1	40	17.7%	-5.50 [-9.12, -1.88]	4 weeks	3mg
Vidor 2013	21.3	18.2	16	38	20.5	16	6.0%	-16.70 [-30.13, -3.27]	4 weeks	5mg
Karateev 2007	54.4	18	17	43.4	26.4	18	5.1%	11.00 [-3.90, 25.90]	3 weeks	3mg
**Total (95% CI)**			**253**			**250**	**100.0%**	**-8.96 [-12.85, -5.08]**		

Heterogeneity: Tau² = 18.79; Chi² = 22.50, df = 9 (P = 0.007); I² = 60%
Test for overall effect: Z = 4.52 (P < 0.00001)

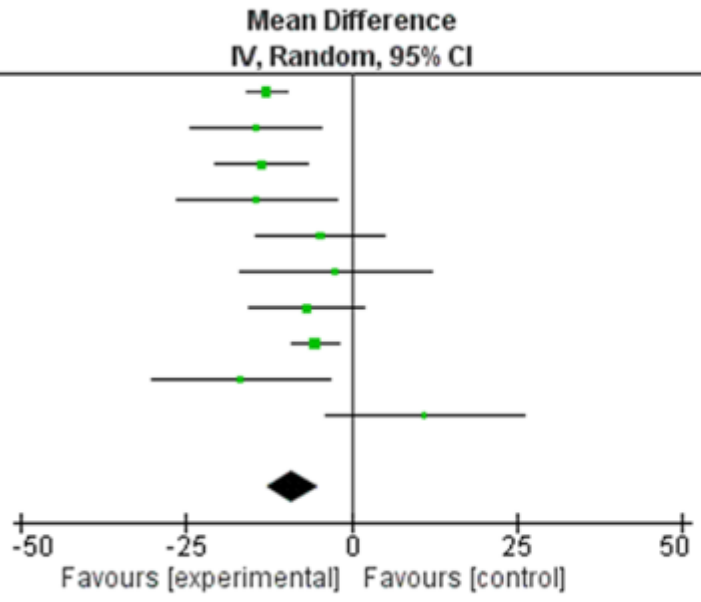

Figure S9 Studies ordered by number of administration (postoperative MSK pain)

Study or Subgroup	Melatonin Mean	Melatonin SD	Melatonin Total	Control Mean	Control SD	Control Total	Weight	Mean Difference IV, Random, 95% CI	Number of Administration	Dosage
LeBrun 2024	36.2	23.8	73	35.6	23	67	3.7%	0.60 [-7.15, 8.35]	42 times	5mg
Ashokkumar 2024	8.4	3.7	32	11.2	3.3	32	8.7%	-2.80 [-4.52, -1.08]	15 times	10mg
Haider 2024	46	24	64	42	24	64	3.3%	4.00 [-4.32, 12.32]	14 times	5mg
Abdelrahman 2020	15	7.4	40	20	0.01	40	8.2%	-5.00 [-7.29, -2.71]	4 times	5mg
Kirksey 2015	20.526	27.381	19	24.211	24.566	19	1.2%	-3.68 [-20.23, 12.86]	7 times	5mg
Fan 2017	31	2	69	32	2	70	9.3%	-1.00 [-1.66, -0.34]	7 times	1mg
Jouybar 2022	31.2	16.3	33	18.5	7.5	33	4.7%	12.70 [6.58, 18.82]	single dose	10mg
Jouybar 2022	31.2	16.3	33	25.6	10.7	33	4.4%	5.60 [-1.05, 12.25]	single dose	10mg
Baradari 2022	19	24	20	31.5	25	20	1.3%	-12.50 [-27.69, 2.69]	single dose	10mg
Esmat 2016	32.8	4.5	25	38	6.9	25	7.4%	-5.20 [-8.43, -1.97]	single dose	7mg
Esmat 2016	32.8	4.5	25	32.8	4.5	25	8.1%	0.00 [-2.49, 2.49]	single dose	7mg
Javaherforooshzadeh 2018	18	5.8	30	10	3.7	30	8.1%	8.00 [5.54, 10.46]	single dose	6mg
Modir (a) 2022	41.6	4.61	30	42.3	4.3	30	8.3%	-0.70 [-2.96, 1.56]	single dose	6mg
Modir (b) 2022	54.7	5.7	30	52.3	4.3	30	8.0%	2.40 [-0.15, 4.95]	single dose	6mg
Modir (b) 2022	54.7	5.7	30	55.7	5	30	7.9%	-1.00 [-3.71, 1.71]	single dose	6mg
Modir (b) 2022	54.7	5.7	30	52.3	6.3	30	7.6%	2.40 [-0.64, 5.44]	single dose	6mg
**Total (95% CI)**			**583**			**578**	**100.0%**	**0.54 [-1.35, 2.44]**		

Heterogeneity: Tau² = 9.99; Chi² = 111.05, df = 15 (P < 0.00001); I² = 86%
Test for overall effect: Z = 0.56 (P = 0.57)

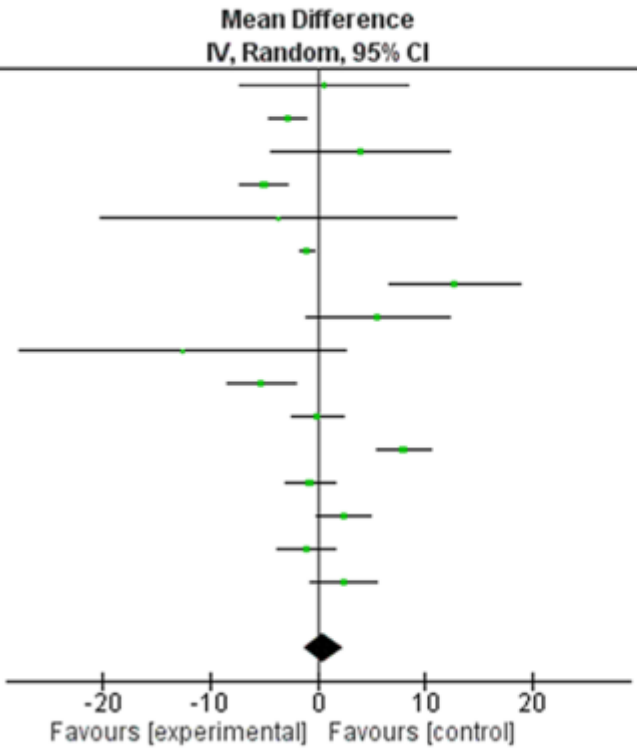

Figure S10. Funnel plot of surgical conditions

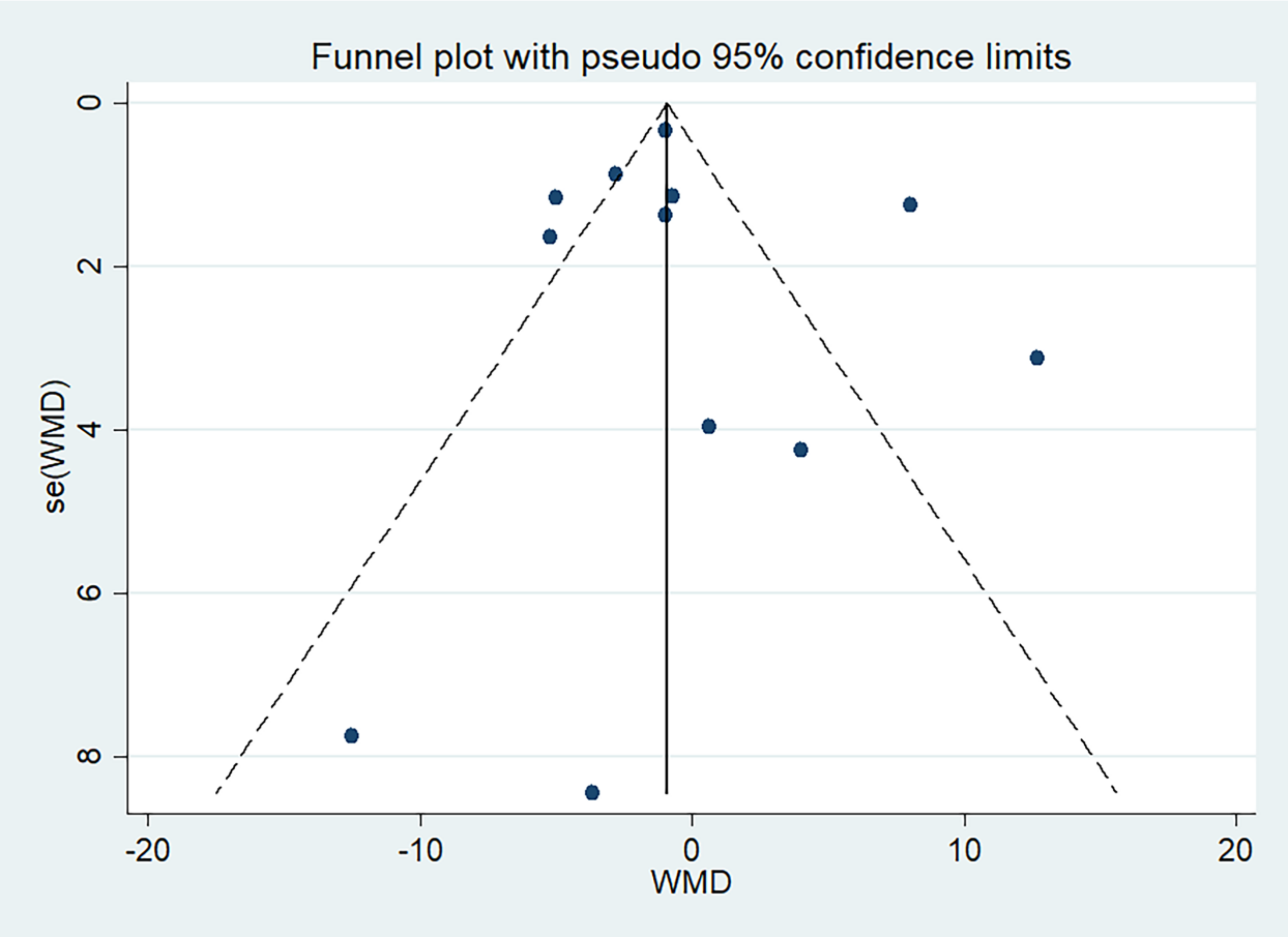

Table S1. Study Characteristics.

References	Country	Condition (n; female%)	Mean age (SD)	Study arms		Treatment duration	Instruments	
				Intervention (n)	Control (n)		Pain intensity	Sleep quality
Karateev et al, 2007[44]	Russia	Rheumatoid arthritis (38; 100.0)	49.3 (12.0)	Melatonin 3 mg/day (17)	Placebo once/day (18)	3 weeks	VAS (0-100)	Questionnaire developed by research group (0-24)
Hussain et al, 2011[41]	Iraq	Fibromyalgia (101; 94.1)	38.8 (11.2)	Melatonin 5mg/day (27)	Fluoxetine 20mg/day (24)	8 weeks	Fibromyalgia Impact Questionnaire-Pain subscale (0-10)	Fibromyalgia Impact Questionnaire-Rest/Sleep subscale (0-10)
Vidor et al, 2013[50]	Brazil	Temporomandibular disorders (32; 100.0)	30.9 (4.8)	Melatonin 5mg/day (16)	Placebo 5mg/day (16)	4 weeks	VAS (0-10)	VASQS (0-10)
de Zanette et al, 2014[37]	Brazil	Fibromyalgia (63;100.0)	49.0 (8.0)	Melatonin 10mg/day (21)	Amitriptyline 25mg/day (21)	6 weeks	VAS (0-100)	PSQI
Kirksey et al, 2015[22]	USA	Total knee arthroplasty (50; 54.1)	65.8 (12.6)	Melatonin 5mg/day (19)	Placebo 5mg/day (18)	7 days	VAS (0-10)	Sleep quality scale (0-10)

References	Country	Condition (n; female%)	Mean age (SD)	Study arms		Treatment duration	Instruments	
				Intervention (n)	Control (n)		Pain intensity	Sleep quality
Esmat & Assim, 2016[39]	Egypt	Lumbar laminectomy (75; 44.0)	53.6 (4.7)	Group 1: Melatonin 7mg (25)<br>Group 2: Fentanyl 50 μg/h (n=25)	Placebo (25)	14 h (patch applied 2 h pre-operation, removed 12 h post-operation)	VAS (0-10)	-
Kurganova & Danilov, 2016[45]	Russia	Chronic nonspecific back pain (178; 78.1)	51.8 (6.0)	Comparison 1: Artra (glucosamine hydrochloride 500 mg and chondroitin sulfate 500 mg as tablets) twice a day during the first month, and then one tablet daily during the next two months + Melaxen (melatonin 3 mg/day) at night (31)	Comparison 1: Artra (glucosamine hydrochloride 500 mg and chondroitin sulfate 500 mg as tablets) twice a day during the first month, and then one tablet daily during the next two months (29)	3 months	VAS (0-10)	PSQI (0-21)

References	Country	Condition (n; female%)	Mean age (SD)	Study arms		Treatment duration	Instruments	
				Intervention (n)	Control (n)		Pain intensity	Sleep quality
				Comparison 2: Artra one tablet twice daily and diclofenac 25 mg 2-3 times daily + Melaxen (melatonin) 3mg/day (30)	Comparison 2: Artra one tablet twice daily and diclofenac 25 mg 2-3 times daily (30)			
				Comparison 3: Diclofenac 25 mg three times/day and Melaxen 3mg/day (29)	Comparison 3: Diclofenac 25 mg three times daily (29)			
Fan et al, 2017[40]	China	Hip arthroplasty (148; 61.9)	74.5 (5.5)	Melatonin 1mg (69)	Placebo 1mg (70)	7 days (1 day preoperatively and 5 days postoperatively)	VAS (0-10)	VAS (0-100)
Javaherforooshzadeh et al, 2018[42]	Iran	Lumbar spine surgery (90; 53.3)	47.3 (5.5)	Melatonin 6mg (30)	Gabapentin 600mg (30)	Single dose; 100 minutes before	VAS (0-10)	-

References	Country	Condition (n; female%)	Mean age (SD)	Study arms		Treatment duration	Instruments	
				Intervention (n)	Control (n)		Pain intensity	Sleep quality
						induction of anesthesia		
Altiparmak et al, 2019[33]	Turkey	Neuropathic pain (80; 48.8)	48.3 (13.3)	Melatonin 3mg + Gabapentin 300mg at Day1/ 600mg at Day2/ 900mg at Day3 and after (40)	Placebo 3mg + Gabapentin 300mg at Day1/ 600mg at Day2/ 900mg at Day3 and after (40)	30 days	Verbal Raing Scale (0-10)	PSQI (0-21)
Abdelrahman et al, 2020[32]	Egypt	Bilateral multiple fracture ribs (80; 18.8)	25.2 (1.2)	Melatonin 5mg (40)	Placebo 5mg (40)	1 hour before thoracic epidural insertion and then every 12 hours till the cessation of bupivacaine infusion	VAS (0-10)	Patient reported sleep quality
Baradari et al, 2022[35]	Iran	Lumbar disc surgery (79; 32.5)	39.8 (8.0)	Melatonin 10mg (20)	Placebo (20)	Single Dose; One hour prior to surgery	VAS (0-10)	-

References	Country	Condition (n; female%)	Mean age (SD)	Study arms		Treatment duration	Instruments	
				Intervention (n)	Control (n)		Pain intensity	Sleep quality
Clarkson et al, 2022[36]	USA	Primary total joint arthroplasty (118; 49.2)	64.5 (8.0)	Melatonin 6 mg (50)	Placebo (53)	6 weeks	-	PSQI (0-21)
Jouybar et al, 2022[43]	Iran	Laminectomy (99; 67.7)	48.84 (10.26)	Group 1: Melatonin 10mg (two 5mg tablets) of melatonin (33)	Group2: Dexmedetomidine 1µg/kg (33)<br>Group3: Gabapentin 600mg (two 300mg tablets) (33)	Single dose; two hours before the operation	VAS (0-10)	-
Modir et al, 2022a[48]	Iran	Intervertebral disc herniation surgery (60; 50.0)	39.1 (6.9)	Melatonin 6 mg (30)	Zolpidem 10 mg (30)	Single dosage; 90 minutes before surgery	VAS (0-10)	-
Modir et al, 2022b[47]	Iran	Hip fracture surgery (120; 46.7)	39.5 (11.1)	Group 1: Melatonin 6mg (30)<br>Group 2: Dextromethorphan 1mg/kg (30)	Placebo, same amount of distilled water (30)	Single dosage; 90 minutes before surgery	VAS (0-10)	-

References	Country	Condition (n; female%)	Mean age (SD)	Study arms		Treatment duration	Instruments	
				Intervention (n)	Control (n)		Pain intensity	Sleep quality
				Group 3: Gabapentin 600 mg (30)				
Palimi et al, 2022[49]	Iran	Rheumatoid arthritis, (70; 82.8)	42.7 (7.0)	Melatonin 3mg/day (32)	Placebo (32)	60 days	VAS (0-100)	PSQI (0-21)
Delkash et al, 2023[38]	Iran	Knee osteoarthritis (57; 63.2)	64.9 (3.9)	Melatonin 3 mg/day, titrated up to 10 mg (28)	Duloxetine 60 mg/day, titrated up to 120 mg as needed (29)	3 months	WOMAC-Pain (0-20)	-
Shodiev et al, 2023[21]	Russia	Knee osteoarthritis, (36; 91.7)	60.0 (4.5)	Melatonin 3mg/day + conservative treatment (12)	Group 1: conservative treatment (12)<br>Group 2: Healthy control (12)	30 days	KOOS-Pain (0-100)	PSQI (0-21)
Ashokkumar et al, 2024[34]	India	Zygomatico maxillary complex fractures (64; 10.9)	37.6 (12.3)	Melatonin 10mg/day (32)	Placebo 10mg/day (32)	15 days	VAS (0-5)	-
Haider et al, 2024[23]	USA	Total knee arthroplasty (138; 69.5)	65.5 (11.8)	Melatonin 5 mg/day (64)	Placebo 5mg/day (64)	14 days	VAS (0-10)	PROMIS sleep disturbance
LeBrun et al, 2024[46]	USA	Total knee arthroplasty	67.3 (8.7)	Melatonin 5mg/day (73)	Placebo 125mg/day (67)	6 weeks	VAS (0-10)	PSQI (0-21)

References	Country	Condition (n; female%)	Mean age (SD)	Study arms		Treatment duration	Instruments	
				Intervention (n)	Control (n)		Pain intensity	Sleep quality
		(172; 55.2)						
Perez et al, 2024[29]	USA	Arthroscopic rotator cuff repair surgery (80; 28.8)	61.0 (7.6)	Melatonin 5mg/day (40)	Sleep hygiene recommendations (40)	6 weeks	-	PSQI (0-21)

Abbreviations: KOOS: Knee Injury and Osteoarthritis Outcome Score; PROMIS: Patient-Reported Outcomes Measurement Information System; PSQI: Pittsburgh Sleep Quality Index; VAS: Visual Analogue Scale; VASQS: VAS Quality of Sleep (Visual analogue scale quality of sleep); WOMAC: Western Ontario and McMaster Universities Osteoarthritis Index
Legend: Participants column shows the number of patients with the musculoskeletal condition and percentage of women, followed by mean age (standard deviation). Intervention and control columns list treatment names with dosage (if reported) and sample size. Scoring Instrument column lists measurement scales with their ranges, when available.

Table S2. Univariable meta-regression analyses for chronic MSK pain and postoperative pain.

Category	Variable	Estimate (SE)[a]	t	P value	95% CI	tau^2	I^2res	Adj R^2
**Chronic MSK pain**								
	Dose	0.8320 (1.3235)	0.63	0.547	−2.2192 to 3.8849	15.82	62.88%	−4.39%
	Duration	−0.9709 (0.2803)	−3.46	0.009	−1.6173 to −0.3245	0	11.14%	100.00%
**Postoperative pain**								
	Dose	0.3781 (0.5546)	0.68	0.506	−0.8113 to 1.5676	19.6	87.27%	−5.55%
	Number of administrations	−0.0751 (0.1424)	−0.53	0.606	−0.3806 to 0.2304	19.13	85.00%	−3.01%

Meta-regression analyses were performed using REML, estimation with the Knapp–Hartung modification.
Abbreviations: SE, standard error; tau^2, residual between-study variance; I^2res, residual heterogeneity; Adj R^2, adjusted proportion of between-study variance explained.
Dose was coded in milligrams (mg), duration in weeks, and number of administrations as the total number of administrations.
